# The spectral evolution of white dwarfs: where do we stand?

**DOI:** 10.1007/s10509-024-04307-5

**Published:** 2024-04-26

**Authors:** Antoine Bédard

**Affiliations:** Department of Physics, University of Warwick, Coventry, CV4 7AL UK

**Keywords:** White dwarf stars (1799), Atmospheric composition (2120), Stellar evolution (1599)

## Abstract

White dwarfs are the dense, burnt-out remnants of the vast majority of stars, condemned to cool over billions of years as they steadily radiate away their residual thermal energy. To first order, their atmosphere is expected to be made purely of hydrogen due to the efficient gravitational settling of heavier elements. However, observations reveal a much more complex situation, as the surface of a white dwarf (1) can be dominated by helium rather than hydrogen, (2) can be polluted by trace chemical species, and (3) can undergo significant composition changes with time. This indicates that various mechanisms of element transport effectively compete against gravitational settling in the stellar envelope. This phenomenon is known as the spectral evolution of white dwarfs and has important implications for Galactic, stellar, and planetary astrophysics. This invited review provides a comprehensive picture of our current understanding of white dwarf spectral evolution. We first describe the latest observational constraints on the variations in atmospheric composition along the cooling sequence, covering both the dominant and trace constituents. We then summarise the predictions of state-of-the-art models of element transport in white dwarfs and assess their ability to explain the observed spectral evolution. Finally, we highlight remaining open questions and suggest avenues for future work.

## Introduction

White dwarfs are the end products of the life cycle of the wide majority of stars and, as such, contain a wealth of information on the history of the Milky Way. The archetypical white dwarf has a mass of $\simeq 0.60\,M_{\odot}$ within a radius of only $\simeq 0.013\,R_{\odot}$, resulting in an extremely high density. The stellar interior is thus strongly electron-degenerate, giving rise to a peculiar but well-defined (and hence very useful) mass–radius relation. Furthermore, the intense gravitational field results in a highly stratified chemical structure: a large carbon–oxygen core, a thin helium mantle, and an even thinner hydrogen layer. The helium and hydrogen shells making up the stellar envelope are generally thought to account for $\simeq 10^{-2}$ and $\simeq 10^{-4}$ of the total mass. The tenuous observable atmosphere, comprising a mere $\simeq 10^{-14}$ of the total mass, is usually composed of hydrogen to a high degree of purity due to the efficient gravitational settling of heavier elements. Because nuclear burning no longer occurs in their interior, white dwarfs continuously cool and fade over time as they slowly radiate away their residual thermal energy. The effective temperature decreases from $\simeq 100{,}000$ K to $\simeq 3000$ K in about 10 Gyr, thereby serving as a direct proxy for the cooling age. Thanks to this unique property, white dwarfs act as reliable cosmic clocks, routinely used to measure the age of stellar populations and the stellar formation history of the Milky Way (Winget et al. 1987; Oswalt et al. 1996; Leggett et al. 1998; Fontaine et al. 2001; Hansen et al. 2007; García-Berro et al. 2010; Kalirai 2012; Tremblay et al. 2014; Kilic et al. 2017; Fantin et al. 2019; Isern 2019; Cukanovaite et al. 2023).

Given these basic characteristics, white dwarfs are often viewed as very simple astrophysical objects. However, upon closer scrutiny, one is faced with the paradox that the applications arising from their relative simplicity require a detailed understanding of their structure and evolution, which in turn involves many complex considerations. Building an accurate model of a white dwarf requires a multitude of microphysical ingredients (equation of state, radiative and conductive opacity, diffusion coefficients, etc.) over an exceptionally wide range of conditions, including some highly challenging physical regimes (Saumon et al. 2022). Moreover, the cooling process is not as uneventful as one may naively believe: it is markedly impacted by phenomena as diverse as residual nuclear burning, neutrino emission, core crystallisation, and convective coupling (Fontaine et al. 2001; Althaus et al. 2010; Chen et al. 2021). The cooling rate also depends on the masses of the hydrogen and helium layers as well as on the relative amounts of carbon and oxygen in the core, which are dictated by complex (and still poorly understood) mechanisms taking place in previous evolutionary stages (Fontaine et al. 2001; Althaus et al. 2010). In hindsight, exploiting the potential of stellar remnants as cosmic clocks proves to be a highly non-trivial endeavour.

One particularly intricate and surprising property of white dwarfs as a group is the diverse and changing nature of their surface composition. Although most objects possess a standard hydrogen atmosphere, many instead exhibit a helium atmosphere, indicating that they have lost the bulk of their outer hydrogen layer in an earlier evolutionary phase. Furthermore, in both cases, it is not uncommon for elements other than the main constituent to be present in small amounts, despite the expected efficiency of gravitational settling. Even more puzzling is the fact that the surface composition of white dwarfs can change with time as they cool, a phenomenon referred to as spectral evolution. These variations involve not only the abundances of the trace contaminants, but also the nature of the dominant constituent, with atmospheres transitioning from helium-rich to hydrogen-rich and vice versa. This suggests that the transport of elements in the envelope of white dwarfs is in fact much more complicated than a simple sedimentation process. Indeed, additional transport mechanisms such as convection, winds, and accretion may effectively compete against gravitational settling and thus alter the chemical makeup of the observable layers (Fontaine and Wesemael 1987).

The study of spectral evolution can reveal valuable information on the envelope of white dwarfs, which in turn has crucial implications in various areas of astrophysics. As mentioned above, the envelope composition influences the cooling rate and must therefore be well constrained to ensure the reliability of stellar age-dating applications (Fontaine et al. 2001). White dwarf envelopes also bear the chemical imprint of complex physical processes occurring in their progenitors and can thus help improve our knowledge of previous phases of stellar evolution (Werner and Herwig 2006). Moreover, as we will see later, spectral evolution finds a remarkable application in the field of exoplanets as it can provide unique information on the internal structure of these objects (Jura and Young 2014). Finally, the changing surface composition of a white dwarf represents a direct window on the physics of element transport in stars (Salaris and Cassisi 2017).

The spectral evolution of white dwarfs has been a topic of active research for several decades, with efforts directed along two main lines of investigation. The first approach aims to provide an empirical characterisation of the phenomenon, that is, to constrain the observed variations in atmospheric composition along the cooling sequence. This undertaking rests mostly on the analysis of observational data from large astronomical surveys; in particular, key advances have been made possible by the Sloan Digital Sky Survey (York et al. 2000) and, more recently, the Gaia mission (Gaia Collaboration et al. 2016). The second approach is theoretical in nature and seeks to identify and understand the physical mechanisms giving rise to spectral evolution. This involves the calculation of white dwarf models considering the effects of various element transport processes, the predictions of which can then be compared to empirical constraints. These two fields are complementary and have often driven one another, with new observational findings motivating further modelling efforts and vice versa. The ultimate goal is to develop a complete theory of spectral evolution, offering a consistent explanation for all the observed features (Fontaine and Wesemael 1987).

The purpose of this review is to provide an exhaustive picture of our current understanding of white dwarf spectral evolution, highlighting recent advances and remaining challenges. The paper is structured in two main parts relating to the two approaches outlined above: Sect. 2 focuses on the empirical evidence, while Sect. 3 describes the theoretical interpretation. Finally, concluding remarks are given in Sect. 4.

## Empirical evidence

Except in a few rare cases mentioned below, the spectral evolution of white dwarfs is usually not witnessed in real time. Rather, the existence of this phenomenon is deduced from the fact that large samples of white dwarfs show statistical variations of surface composition as a function of effective temperature (and thus as a function of cooling age). By studying these variations, one can infer how the atmospheric composition of individual objects changes with time along the cooling sequence. This is the empirical approach to spectral evolution, where one attempts to constrain the net effect of the various element transport mechanisms on the observable outer layers. As such an endeavour rests heavily on the study of white dwarf spectral types and atmospheric properties, we first review some basic notions of spectral classification and analysis. We then provide a comprehensive overview of the latest findings regarding the surface composition of white dwarfs, in terms of both the dominant constituent and the trace contaminants.

### Preliminaries: spectral classification and atmospheric characterisation

The standard spectral classification system for white dwarfs is described in detail in Sion et al. (1983) and Wesemael et al. (1993). Spectral types always begin with the letter D (to indicate the degenerate nature of the star) and include a second letter identifying the most prominent lines in the optical region of the spectrum. The main spectral classes and their associated features are as follows:

- DA: hydrogen features;
- DB: neutral helium features;
- DC: no features (continuous spectrum);
- DO: ionised helium features;
- DQ: carbon features;^1^
- DZ: other metal features.

**Fig. 1:**
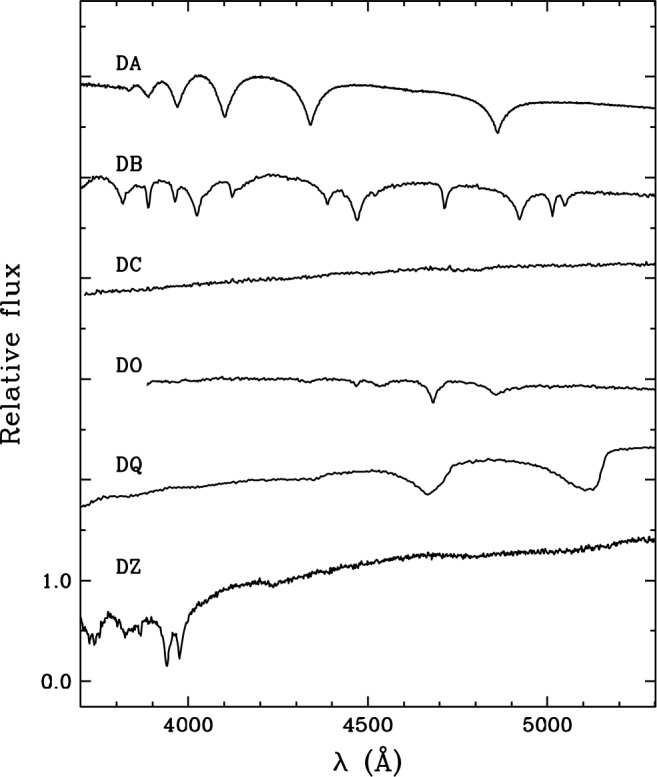
Examples of optical spectra of DA, DB, DC, DO, DQ, and DZ white dwarfs. The spectra are normalised at $\lambda = 4200$ Å and shifted vertically from each other by an arbitrary amount. The data are taken from Bergeron et al. (1997), Bergeron et al. (2011), Giammichele et al. (2012), and Subasavage et al. (2017)

Figure 1 shows examples of white dwarf optical spectra for each of these classes.

The spectral type is a rough indicator of the surface composition but does not always reflect the nature of the main chemical constituent. For instance, a white dwarf with a pure-hydrogen atmosphere is indeed of the DA type over most of the sequence cooling, but is of the DC type at $T_{\mathrm{eff}} \lesssim 5000$ K because hydrogen transitions are not excited at these low temperatures. Likewise, a white dwarf with a pure-helium atmosphere is classified as DO at $T_{\mathrm{eff}} \gtrsim 45{,}000$ K, DB at $45{,}000\,\mathrm{K} \gtrsim T_{\mathrm{eff}} \gtrsim 11{,}000$ K, and DC at $T_{\mathrm{eff}} \lesssim 11{,}000$ K. The shift from DO to DB is due to the recombination of the helium ions, while the shift from DB to DC results from the disappearance of helium transitions. Finally, the atmosphere of DQ- and DZ-type stars actually consists mostly of helium and contains only small amounts of carbon or other metals. Their spectral appearance is attributable to their low effective temperature ($T_{ \mathrm{eff}} \lesssim 11{,}000$ K): under these conditions, helium is invisible while heavier elements remain visible, even at low abundances.^2^

Additional letters can be appended to indicate the presence of secondary spectral features. For instance, a white dwarf spectrum showing both strong hydrogen lines and weak ionised helium lines is classified as DAO. Among the many possible hybrid types, some of the most common (and thus most relevant for the present review) are DAO, DBA, DAZ, and DBZ. Occasionally, spectral signatures from more than two elements can be detected, giving rise to even more specific classes such as DBAZ.^3^

Although the spectral type is a useful classification label, it is not sufficient in itself to characterise the atmosphere of a white dwarf. Extracting the physical information encoded in spectral observations generally involves two analysis steps: the calculation of atmosphere models from first principles, and a quantitative comparison between the models and data. This allows the determination of the atmospheric parameters of a given star, namely, its effective temperature, surface gravity, and atmospheric composition. This procedure comes in two main variants, the so-called spectroscopic and photometric techniques, differing in the kind of observational data being analysed. The spectroscopic technique relies on the detailed shape of the spectral lines and thus requires a relatively high signal-to-noise spectrum (Bergeron et al. 1992; Finley et al. 1997; Liebert et al. 2005; Bergeron et al. 2011), while the photometric technique uses the overall spectral energy distribution built from apparent magnitudes together with a trigonometric parallax measurement (Bergeron et al. 1997, 2001, 2019; Gentile Fusillo et al. 2019). The photometric method has the advantage of being more widely applicable (as it does not require spectroscopic observations) but the inconvenience of being much less sensitive to the surface composition (which is thus often assumed rather than inferred). The measured atmospheric parameters can be combined to theoretical mass-radius relations and cooling calculations to derive other stellar properties of interest, such as the mass, radius, luminosity, and cooling age (Renedo et al. 2010; Camisassa et al. 2016; Bédard et al. 2020; Salaris et al. 2022; Bauer 2023).

### The main constituent: hydrogen or helium?

Despite their rich diversity of spectral appearances, nearly all white dwarfs fall into one of two main categories: those with a hydrogen-dominated atmosphere, and those with a helium-dominated atmosphere. Therefore, the most basic questions to be answered are: what are the respective proportions of these two groups among the white dwarf population, and how do these proportions change along the cooling sequence? In practice, a common strategy consists in determining the atmospheric parameters of a large number of white dwarfs and calculating the fraction of helium-rich objects as a function of effective temperature. This simplified approach, where one focuses solely on the dominant chemical constituent and ignores the possible presence of trace elements, provides a first-order picture of spectral evolution. The assessment of the helium-atmosphere fraction has been an active research topic for several decades (Sion 1984; Fleming et al. 1986; Greenstein 1986; Fontaine and Wesemael 1987; Dreizler and Werner 1996; Bergeron et al. 1997; Napiwotzki 1999; Bergeron et al. 2001; Eisenstein et al. 2006; Tremblay and Bergeron 2008; Krzesinski et al. 2009; Bergeron et al. 2011; Giammichele et al. 2012; Reindl et al. 2014a; Limoges et al. 2015).

In recent years, the number, scope, and quality of such studies have significantly increased thanks to the advent of the Gaia mission (Gaia Collaboration et al. 2016). In 2018, the second data release of Gaia (Gaia Collaboration et al. 2018b) provided, for the first time, exquisite astrometry and photometry for more than 250,000 high-confidence white dwarf candidates (Gentile Fusillo et al. 2019). This represented an almost tenfold increase in the number of known white dwarfs (Kepler et al. 2019) and a thousandfold increase in the number of available parallax measurements (Bédard et al. 2017). The use of Gaia parallaxes in conjunction with optical photometry, either from Gaia or other large surveys such as the Sloan Digital Sky Survey (SDSS; York et al. 2000) and the Panoramic Survey Telescope and Rapid Response System (Pan-STARRS; Chambers et al. 2016), enabled the characterisation of white dwarf atmospheres on an unprecedented scale (Jiménez-Esteban et al. 2018; Bergeron et al. 2019; Genest-Beaulieu and Bergeron 2019a; Gentile Fusillo et al. 2019; Ourique et al. 2019; Tremblay et al. 2019). Nevertheless, as mentioned above, these photometric analyses are usually insensitive to the surface composition, which is consequently assumed rather than derived. For spectral evolution studies, additional information is needed to unambiguously determine the nature of the dominant atmospheric constituent, a requirement that necessarily limits the size of the usable sample.

The most direct and reliable option is, of course, spectroscopy. Genest-Beaulieu and Bergeron (2019b), Ourique et al. (2019), and Bédard et al. (2020) made use of SDSS spectroscopy, which remains at the time of writing the largest available source of medium-resolution white dwarf spectra (nearly 40,000; Kepler et al. 2019, 2021). Blouin et al. (2019b) and Caron et al. (2023) focused on cool white dwarf samples ($T_{\mathrm{eff}} \lesssim 10{,}000$ K) built from a mix of SDSS and archival spectroscopic data from various sources. McCleery et al. (2020) and O’Brien et al. (2024) relied on the nearly complete spectroscopic follow-up of white dwarfs in the local 40 pc volume. At intermediate temperatures ($25{,}000\,\mathrm{K} \gtrsim T_{\mathrm{eff}} \gtrsim 10{,}000$ K), an alternative possibility is the use of ultraviolet or narrow-band blue photometry, which is sensitive to the presence or absence of the Balmer jump and thus allows to discriminate between hydrogen-rich and helium-rich atmospheres. Cunningham et al. (2020) and López-Sanjuan et al. (2022) exploited this method using data from the Galaxy Evolution Explorer (GALEX; Martin et al. 2005) and the Javalambre Photometric Local Universe Survey (J-PLUS; Cenarro et al. 2019), respectively. Among all these studies, those that rely on SDSS observations tend to have a larger sample size and/or a broader $T_{\mathrm{eff}}$ coverage, but suffer from complex selection biases. The other works typically trade off lower-number statistics for a better completeness.

More recently, the third data release of Gaia (Gaia Collaboration et al. 2021, 2023b) increased the number of high-confidence white dwarf candidates to more than 350,000 (Gentile Fusillo et al. 2021). Most importantly for the purpose of this review, it also provided low-resolution spectra for about 100,000 of these objects (Gaia Collaboration et al. 2023a), thereby producing the largest sample so far for which a rough estimate of the surface composition can be made. Jiménez-Esteban et al. (2023), Torres et al. (2023), and Vincent et al. (2024) leveraged this new data set to measure the helium-atmosphere fraction across a broad $T_{\mathrm{eff}}$ range with improved precision and/or completeness (depending on the adopted sample). However, the accuracy of these results may still suffer from systematic classification errors due to the very low resolution of the Gaia spectra (García-Zamora et al. 2023; Vincent et al. 2024).

**Fig. 2:**
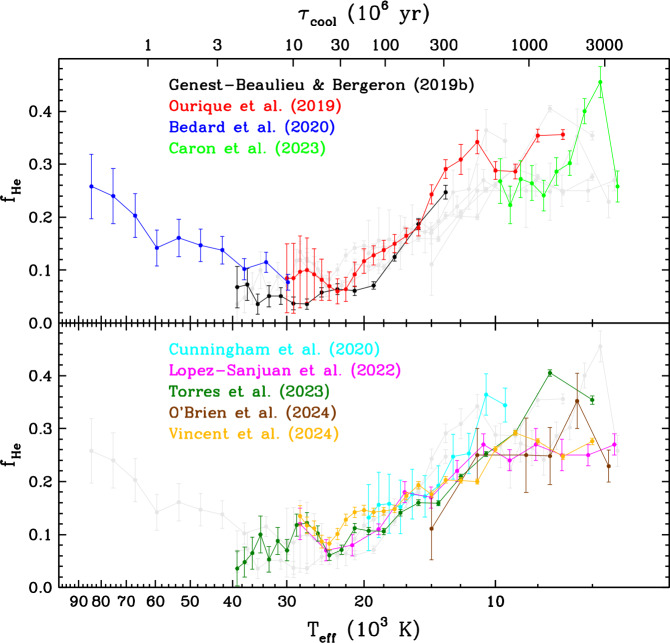
Fraction of helium-atmosphere white dwarfs as a function of effective temperature in various post-Gaia studies. For clarity, the studies are arbitrarily divided into those that rely on SDSS spectroscopy (top panel) and those that do not (bottom panel). The curves emphasised in one panel are displayed in light grey in the other panel to allow comparison. Values at $T_{\mathrm{eff}} > 90{,}000$ K and $T_{\mathrm{eff}} < 5000$ K have been purposefully excluded because they are considered unreliable. The results of Blouin et al. (2019b), McCleery et al. (2020), and Jiménez-Esteban et al. (2023) are not shown as they have been superseded more recently by those of Caron et al. (2023), O’Brien et al. (2024), and Torres et al. (2023), respectively. For reference, the top axis gives the cooling age of a standard 0.60 $M_{\odot }$ hydrogen-rich white dwarf according to the theoretical evolutionary calculations of Bédard et al. (2020)

Figure 2 shows the fraction of helium-dominated white dwarfs as a function of effective temperature as determined in these works. Let us first focus on the global behaviour of the function at $T_{\mathrm{eff}} \gtrsim 10{,}000$ K, where the agreement between different papers is most satisfactory. At the beginning of the cooling sequence, 20–30% of white dwarfs have a helium-rich atmosphere.^4^ This proportion then gradually decreases with decreasing temperature and reaches a minimum value of 5–15%, which subsequently remains roughly constant within the range $40{,}000\,\mathrm{K} \gtrsim T_{\mathrm{eff}} \gtrsim 20{,}000$ K. This part of the cooling sequence is historically known as the DB gap, in reference to the scarcity of helium-atmosphere DB white dwarfs relative to their hydrogen-atmosphere DA counterparts (Fontaine and Wesemael 1987). At lower temperatures, the helium-rich fraction gradually re-increases and reaches 20–35% at $T_{\mathrm{eff}} \simeq 10{,}000$ K, approximately the same value as for very hot white dwarfs.

This V-shaped function tells a compelling story: the surface composition of some objects must evolve from helium to hydrogen and then back to helium as they cool. More specifically, Fig. 2 suggests that there exist three distinct evolutionary channels among the white dwarf population at $T_{\mathrm{eff}} \gtrsim 10{,}000$ K:

1. stars that have and retain a hydrogen atmosphere (DA type);
2. stars that have and retain a helium atmosphere (DO then DB type);
3. stars that initially have a helium atmosphere (DO type) but then experience a helium-to-hydrogen (DO-to-DA) transition at high temperature and a hydrogen-to-helium (DA-to-DB) transition at low temperature.

From the extrema of the helium-dominated fraction quoted above, we can deduce that these three channels account for about 75, 10, and 15% of the white dwarf population, respectively. These approximate numbers arise from the fact that the fraction of hydrogen-rich objects is always at least about 75% while the fraction of helium-rich objects is always at least about 10%.

Although this interpretation successfully explains the overall variation of the helium-rich fraction, some details remain unclear. The slope of the decrease at high temperatures is uncertain, as there is only one modern study in this domain and it has large error bars. The same can be said of the increase at lower temperatures but for a different reason: here multiple results are available but they exhibit significant scatter. In particular, note that the curves of Torres et al. (2023) and Vincent et al. (2024) based on Gaia spectrophotometry achieve a very high precision but disagree with each other at the $\simeq 2\sigma $ level over most of this range. Also of interest is the possible presence of substructures in Fig. 2, most notably a small bump in the helium-rich fraction within the DB gap. All in all, more work is needed to clarify the fine points of the spectral evolution function.

At $T_{\mathrm{eff}} \lesssim 10{,}000$ K, the current situation is much less satisfactory, with different papers reporting wildly inconsistent results. While some find that the helium-atmosphere fraction remains roughly constant at 20–30%, others instead find that it sharply increases and then decreases with cooling. Among the latter, the location and amplitude of the spike varies from one study to another; the most dramatic variation is seen in Caron et al. (2023), where the helium-rich fraction reaches almost 50% at $T_{\mathrm{eff}} \simeq 6000$ K. If real, such a feature would imply that some white dwarfs that had previously maintained a hydrogen atmosphere (channel 1 above) develop a helium atmosphere at very low temperature and thereby undergo a DA-to-DC transition. Better constraints on the incidence of this spectral transformation would be highly desirable. However, we point out that the volume-complete sample of O’Brien et al. (2024), which is the least affected by selection biases, shows little evidence of spectral evolution at $T_{\mathrm{eff}} \lesssim 10{,}000$ K.

Finally, note that we chose to limit the results displayed in Fig. 2 to $T_{\mathrm{eff}} > 5000$ K. Although some attempts to probe spectral evolution at lower temperatures have been made (Blouin et al. 2019b; Elms et al. 2022; Caron et al. 2023; Vincent et al. 2024), these results should be taken with caution. In this regime, distinguishing hydrogen-rich and helium-rich atmospheres becomes very challenging, as both hydrogen and helium lines disappear and one must instead rely on subtle differences in the spectral energy distribution. Furthermore, severe problems are known to affect current atmospheric models of very cool white dwarfs and thus to jeopardise the accuracy of the inferred parameters, including the effective temperature (Hollands et al. 2018b; Bergeron et al. 2022; Caron et al. 2023; O’Brien et al. 2024). For these reasons, we refrain from speculating on spectral evolution at $T_{\mathrm{eff}} < 5000$ K.

### Trace elements: hydrogen and helium

Although white dwarf atmospheres appear highly pure compared to other types of stars, they are not perfectly pure: elements other than the main constituent are often present in small amounts. The nature and abundances of these contaminants vary significantly along the cooling sequence, thereby adding a layer of complexity to the problem of spectral evolution. These composition variations, although more subtle than those discussed above, still provide a wealth of information on the transport processes at work in the stellar envelope. The study of trace elements at the surface of white dwarfs requires detailed spectroscopic analyses, which have so far been feasible only for the SDSS sample and a few smaller targeted surveys. We first focus on the objects exhibiting either a hydrogen-dominated, helium-polluted atmosphere or a helium-dominated, hydrogen-polluted atmosphere, which we henceforth refer to as hybrid white dwarfs. Observationally, these compositions can translate into various spectral types, namely, DAO, DOA, DAB, DBA, DA, and DC, depending on the effective temperature and relative elemental abundances.

In terms of physical properties, hybrid white dwarfs can be divided into four main groups occupying different parts of the cooling sequence, with very little overlap between them. These are:

- the hot homogeneous white dwarfs with $T_{\mathrm{eff}} \gtrsim 55{,}000$ K;
- the hot stratified white dwarfs with $55{,}000\,\mathrm{K} \gtrsim T_{\mathrm{eff}} \gtrsim 30{,}000$ K;
- the classical DBA white dwarfs with $30{,}000\,\mathrm{K} \gtrsim T_{\mathrm{eff}} \gtrsim 11{,}000$ K;
- the helium-rich DA white dwarfs with $T_{\mathrm{eff}} \lesssim 11{,}000$ K.

**Fig. 3:**
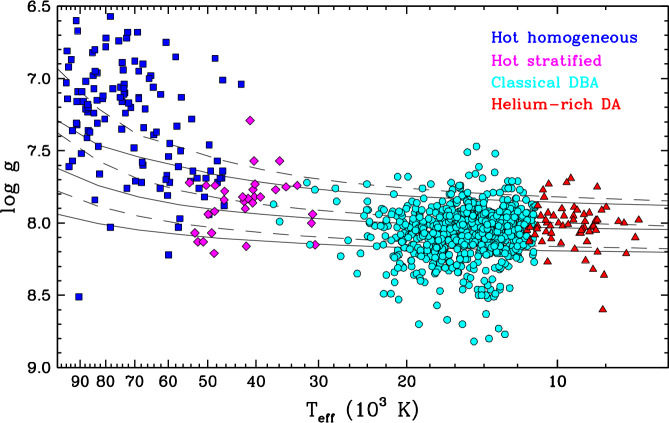
Surface gravity as a function of effective temperature for the four main groups of hybrid-atmosphere white dwarfs: the hot homogeneous stars (blue squares, taken from Gianninas et al. 2010 and Bédard et al. 2020), the hot stratified stars (magenta diamonds, taken from Bédard et al. 2020), the classical DBA stars (cyan circles, taken from Rolland et al. 2018 and Genest-Beaulieu and Bergeron 2019b), and the helium-rich DA stars (red triangles, taken from Rolland et al. 2018 and Coutu et al. 2019). In all cases, objects suspected to be unresolved double white dwarf systems have been excluded. For the stars from Genest-Beaulieu and Bergeron (2019b) and Coutu et al. (2019), the photometric parameters are used and only the objects with a parallax error smaller than 20% are displayed. A small number of objects interpreted as DBA white dwarfs with $T_{\mathrm{eff}} > 30{,}000$ K in Genest-Beaulieu and Bergeron (2019b) do not appear here as they have been reanalysed in Bédard et al. (2020) and found to have either pure-helium or stratified atmospheres. The cool DBA stars from Rolland et al. (2018) that suffer from the spectroscopic high-$\log g$ problem have been excluded. The helium-rich DA stars from Rolland et al. (2018) were assumed to have $\log g = 8.0$ in that paper, hence the $\log g$ values shown here are taken from other sources (Dufour et al. 2017; Gentile Fusillo et al. 2021; Caron et al. 2023). Also displayed for visual guidance are theoretical evolutionary sequences representative of hydrogen-rich (dashed curves) and helium-rich (solid curves) white dwarfs with stellar masses of 0.50, 0.60, and 0.70 $M_{\odot}$ (from top to bottom), taken from Bédard et al. (2020)

Figure 3 shows the location of these objects in the temperature–gravity plane.

In order to discuss the two groups at $T_{\mathrm{eff}} \gtrsim 30{,}000$ K, we must first make a brief digression. Although hybrid white dwarfs obviously contain both hydrogen and helium, the distribution of these elements with depth in the atmosphere is not known a priori. On one hand, the simplest possible configuration is a chemically homogeneous atmosphere, where hydrogen and helium are uniformly mixed. In this case, the surface composition can be parameterised by a conventional elemental abundance, typically the hydrogen-to-helium number ratio, $N_{\mathrm{H}}/N_{\mathrm{He}}$, when hydrogen is trace, and the inverse ratio when helium is trace. On the other hand, given the expected efficiency of gravitational settling in white dwarfs, another plausible configuration is a chemically stratified atmosphere, where a very thin (and thus partially transparent) hydrogen layer floats on top of a helium layer. Because the composition changes with depth, a more natural quantity to describe such an atmosphere is the mass of the hydrogen layer, or rather the ratio of this mass to the total stellar mass, $M_{\mathrm{H}}/M$. As it turns out, the shape of the hydrogen and helium lines is sensitive to the chemical stratification, so a detailed spectroscopic analysis can reveal whether a given star has a homogeneous or stratified atmosphere (Jordan and Koester 1986; Vennes and Fontaine 1992; Bergeron et al. 1994; Barstow and Hubeny 1998; Manseau et al. 2016; Bédard et al. 2020).

Among hot white dwarfs with $T_{\mathrm{eff}} \gtrsim 30{,}000$ K, both the homogeneous and stratified configurations are observed, but in nearly separate regions of the temperature–gravity plane, as shown in Fig. 3. At $T_{\mathrm{eff}} \gtrsim 55{,}000$ K, all known hybrid white dwarfs possess a chemically homogeneous atmosphere. Almost all of them belong to the DAO spectral class, meaning that their surface is dominated by hydrogen. These homogeneous DAO stars are relatively common: overall, they represent about 25–30% of the hot hydrogen-rich white dwarf population (Gianninas et al. 2010; Bédard et al. 2020). This fraction is however strongly temperature-dependent, going from about 50% at $T_{\mathrm{eff}} \simeq 80{,}000$ K to less than 10% at $T_{\mathrm{eff}} \simeq 55{,}000$ K (Bédard et al. 2020). The measured helium abundances span the range $-3.5 \lesssim \log N_{\mathrm{He}}/N_{\mathrm{H}} \lesssim -0.5$, where the lower bound corresponds to the optical detection threshold (Napiwotzki 1999; Good et al. 2004; Gianninas et al. 2010; Tremblay et al. 2011; Bédard et al. 2020; Reindl et al. 2023). This parameter is also strongly correlated with the surface temperature and luminosity, with hotter and brighter objects showing higher levels of helium pollution (Napiwotzki 1999; Gianninas et al. 2010). Finally, hot DAO white dwarfs tend to have lower-than-average stellar masses, as is apparent in Fig. 3, although this may be partially due to systematic errors in spectroscopic analyses (Gianninas et al. 2010; Bédard et al. 2020; Reindl et al. 2023). All in all, these properties indicate that the physical mechanism maintaining helium homogeneously in the outer layers is particularly efficient in hot, low-mass white dwarfs and gradually weakens with cooling.

While hot DAO stars are fairly common, very few of their DOA counterparts are observed, and one may wonder whether helium-dominated, hydrogen-polluted atmospheres are intrinsically rare at the beginning of the cooling sequence. This is likely not the case: the DAO/DOA dichotomy is actually the result of a spectroscopic detection bias. Because ionised helium is a hydrogenic ion, all hydrogen lines are blended with ionised helium lines, which makes it very challenging to detect hydrogen in a hot helium-dominated atmosphere, with a typical visibility limit of $\log N_{\mathrm{H}}/N_{\mathrm{He}} \gtrsim -1.0$ (Werner 1996b; Bédard et al. 2020). Therefore, the hydrogen content of the hot helium-rich white dwarf population is largely unconstrained by spectroscopic observations. Nevertheless, we will see later that this hydrogen content must be nonzero given our current interpretation of spectral evolution.

At $55{,}000\,\mathrm{K} \gtrsim T_{\mathrm{eff}} \gtrsim 30{,}000$ K, most hybrid white dwarfs possess a chemically stratified atmosphere, indicative of efficient gravitational settling. Estimates of the fractional mass of the surface hydrogen layer fall within the range $-18 \lesssim \log M_{\mathrm{H}}/M \lesssim -15$ (Manseau et al. 2016; Bédard et al. 2020). This is simply the range over which both hydrogen and helium lines are detectable; objects with thinner and thicker hydrogen layers appear as pure-helium and pure-hydrogen atmosphere white dwarfs, respectively. As a result of their broad $T_{\mathrm{eff}}$ and $M_{\mathrm{H}}/M$ ranges, stratified white dwarfs exhibit a diverse collection of spectral types: DAO, DOA, DAB, and DBA. Unlike the hotter homogeneous DAO stars, they have average stellar masses, which suggests that these two groups do not share an evolutionary link, as white dwarfs cool at constant mass (Manseau et al. 2016; Bédard et al. 2020).

At $T_{\mathrm{eff}} \lesssim 30{,}000$ K, the hydrogen-rich and helium-rich white dwarf populations are once again characterised by a sharp dichotomy, but a real one this time. While traces of helium in hydrogen-dominated atmospheres are quite rare (Gianninas et al. 2011; Tremblay et al. 2011; Kepler et al. 2019), traces of hydrogen in helium-dominated atmospheres are the rule rather than the exception. In the overwhelming majority of cases, the outer layers are found to be chemically homogeneous. These objects can be further divided into two spectral groups: the classical DBA white dwarfs with $30{,}000\,\mathrm{K} \gtrsim T_{\mathrm{eff}} \gtrsim 11{,}000$ K, and the helium-rich DA white dwarfs with $T_{\mathrm{eff}} \lesssim 11{,}000$ K.

**Fig. 4:**
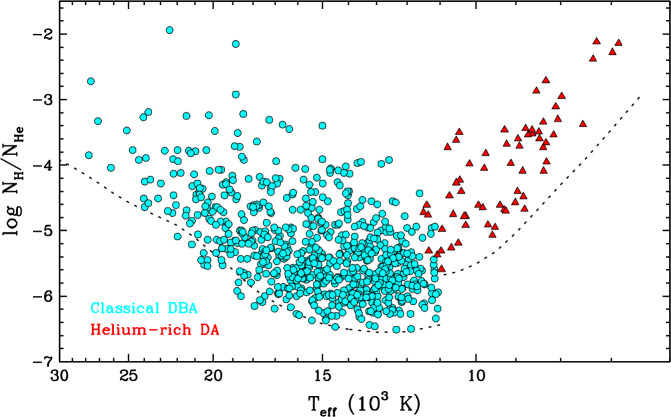
Atmospheric hydrogen abundance (by number relative to helium) as a function of effective temperature for the classical DBA stars and helium-rich DA stars shown in Fig. 3. The dotted lines correspond to the optical spectroscopic detection limit for signal-to-noise ratios of $\simeq 100$ ($T_{\mathrm{eff}} > 11{,}000$ K) and $\simeq 20$ ($T_{ \mathrm{eff}} < 11{,}000$ K), which are representative of the best-observed objects in the DBA and helium-rich DA samples, respectively

The classical DBA stars (so called to distinguish them from the few hotter stratified DBA stars mentioned above) are by far the most numerous and best studied type of hybrid white dwarfs. It is estimated that they represent as much as 60–75% of the helium-dominated population in this part of the cooling sequence (Koester and Kepler 2015; Rolland et al. 2018; Genest-Beaulieu and Bergeron 2019b). Spectroscopic analyses reveal hydrogen abundances in the range $-6.5 \lesssim \log N_{\mathrm{H}}/N_{\mathrm{He}} \lesssim -3.0$, although the lower bound depends on the visibility of the hydrogen lines, which changes with effective temperature (Voss et al. 2007; Bergeron et al. 2011; Koester and Kepler 2015; Rolland et al. 2018; Genest-Beaulieu and Bergeron 2019b; Cukanovaite et al. 2021). Figure 4 shows the hydrogen abundance as a function of effective temperature for a sample of DBA stars. Most objects are found at $T_{\mathrm{eff}} \lesssim 20{,}000$ K and $\log N_{\mathrm{H}}/N_{\mathrm{He}} \lesssim -4.0$. Aside from the hydrogen contamination, the other properties of DBA white dwarfs, such as their mass distribution, are similar to those of their pure-helium DB counterparts (Voss et al. 2007; Bergeron et al. 2011; Genest-Beaulieu and Bergeron 2019b).

At $T_{\mathrm{eff}} \lesssim 11{,}000$ K, helium lines disappear and thus hybrid white dwarfs display a DA-type spectrum, even if hydrogen is not the dominant species. However, the presence of helium can still be inferred from its effect on the H$\alpha $ line, which appears unusually broad and shallow as a consequence of van der Waals interactions (Bergeron et al. 2001; Rolland et al. 2018; Kilic et al. 2020; Caron et al. 2023). Therefore, these so-called helium-rich DA stars can be easily distinguished from standard pure-hydrogen DA stars, and their surface composition can be measured from the shape of the hydrogen lines. The difference between these objects and their hotter DBA siblings is not purely one of spectral appearance: they also tend to have higher hydrogen abundances, as shown in Fig. 4 (Rolland et al. 2018; Coutu et al. 2019; Kilic et al. 2020). This is partially a visibility effect: the hydrogen lines become increasingly difficult to detect with decreasing effective temperature, so cool white dwarfs with a low hydrogen content appear as featureless DC stars. Nevertheless, there is a clear temperature gap between the most hydrogen-polluted members of the two groups, indicating that these objects do not undergo a constant-composition evolution from DBA to helium-rich DA.

Although nearly all hybrid white dwarfs can be assigned to one of the four categories outlined above, a few peculiar objects do not fit any of these typical descriptions. Three well-known examples are PG 1210+533 (Bergeron et al. 1994; Gianninas et al. 2010), HS 0209+0832 (Heber et al. 1997; Wolff et al. 2000), and GD 323 (Koester et al. 1994; Pereira et al. 2005). These stars share a few common characteristics: they are relatively hot ($T_{\mathrm{eff}} \simeq 46{,}000$, 36,000, and 29,000 K, respectively) and their atmosphere appears to be neither perfectly homogeneous nor perfectly stratified. Even more interestingly, the strength of their hydrogen and helium lines is observed to vary on time scales of hours (GD 323) to years (PG 1210+533 and HS 0209+0832). More recently, similar but more extreme variations were discovered in two new peculiar hybrid white dwarfs, one of which successively shows a pure DA and pure DB spectrum over its rotation period of 15 minutes (Caiazzo et al. 2023; Moss et al. 2024). Such horizontal surface inhomogeneities may arise from an atmospheric transformation taking place in the presence of a weak asymmetric magnetic field, and thus these stars may be real-time manifestations of spectral evolution. Besides, we note that a few tens of objects exhibiting a DAB/DBA-type spectrum were actually shown to be unresolved binary systems containing a DA and a DB white dwarf (Wesemael et al. 1994; Bergeron and Liebert 2002; Limoges et al. 2009; Limoges and Bergeron 2010; Bergeron et al. 2011; Tremblay et al. 2011; Genest-Beaulieu and Bergeron 2019b).

### Trace elements: carbon and other metals

Elements heavier than hydrogen and helium are also observed to pollute the surface of many white dwarfs. Among these, carbon has a special status, as it is often detected independently of other metals. We will consequently address carbon-polluted and metal-polluted white dwarfs in turn.

**Fig. 5:**
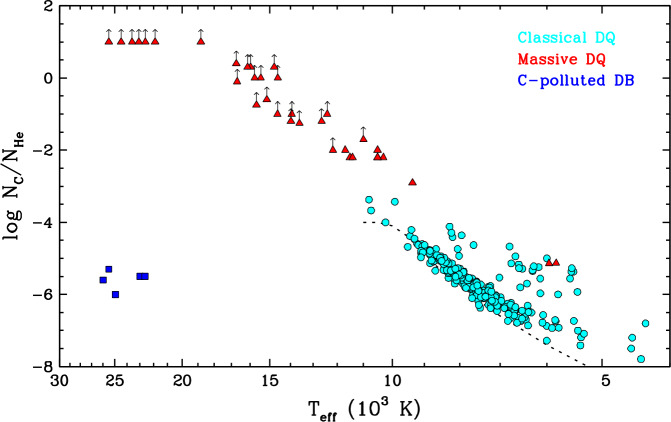
Atmospheric carbon abundance (by number relative to helium) as a function of effective temperature for the three main groups of carbon-bearing white dwarfs: the classical DQ stars (cyan circles, taken from Coutu et al. 2019 and Blouin and Dufour 2019), the massive DQ stars (red triangles, taken from Koester and Kepler 2019, Koester et al. 2020, and Blouin and Dufour 2019), and the carbon-polluted DB stars (blue squares, taken from Petitclerc et al. 2005 and Koester et al. 2014b). An upward-pointing arrow indicates that the displayed carbon abundance is a lower limit rather than a true determination. The classical and massive DQ white dwarfs are arbitrarily defined as having stellar masses lower and higher than 0.80 $M_{\odot}$, respectively. In all cases, objects suspected to be unresolved double white dwarf systems have been excluded. Among the stars in Coutu et al. (2019) and Blouin and Dufour (2019), only those with a parallax error smaller than 20% are displayed. Among the stars in Petitclerc et al. (2005) and Koester et al. (2014b), those that also exhibit traces of other metals have been excluded. The dotted line corresponds to the optical spectroscopic detection limit for a signal-to-noise ratio of $\simeq 20$ ($T_{\mathrm{eff}} < 11{,}000$ K), which is the average value for the cool DQ sample

Most carbon-bearing white dwarfs have a cool ($T_{\mathrm{eff}} \lesssim 10{,}000$ K), helium-dominated atmosphere. Although carbon is merely a trace constituent, the invisibility of helium at low temperatures means that their optical spectrum only exhibits atomic lines and/or molecular bands of carbon, defining the DQ spectral class. These objects are often referred to as the cool DQ or classical DQ stars, and they represent about 20% of the helium-rich population at $10{,}000\,\mathrm{K} \gtrsim T_{\mathrm{eff}} \gtrsim 5000$ K (Bergeron et al. 2019; McCleery et al. 2020; Caron et al. 2023; O’Brien et al. 2024). A remarkable feature of classical DQ white dwarfs is that their carbon contamination is far from random: the surface composition is tightly correlated with the effective temperature, such that cooler objects contain less carbon (Dufour et al. 2005; Koester and Knist 2006; Blouin and Dufour 2019; Coutu et al. 2019; Koester and Kepler 2019; Caron et al. 2023). This is illustrated in Fig. 5, where we reproduce the temperature–abundance diagram of a large DQ sample. The typical carbon abundance sharply decreases from $\log N_{\mathrm{C}}/N_{\mathrm{He}} \simeq -4.0$ at $T_{\mathrm{eff}} \simeq 10{,}000$ K to $\log N_{\mathrm{C}}/N_{\mathrm{He}} \simeq -7.5$ at $T_{\mathrm{eff}} \simeq 5000$ K. The narrowness of the observed sequence is actually a visibility effect: as shown in Fig. 5, the bottom of the sequence perfectly coincides with the optical detection threshold of carbon. This suggests that cool helium-rich atmospheres with lower amounts of carbon do exist but give rise to featureless DC-type optical spectra; and indeed, some DC stars do show carbon lines in the ultraviolet, where the detection limit is lower (Weidemann and Koester 1995; Dufour 2011). Still, the correlation seen in Fig. 5 necessarily implies that the physical mechanism responsible for carbon pollution becomes less effective with cooling. Another interesting property of cool DQ white dwarfs is that they tend to have slightly lower-than-average stellar masses, with a distribution peaking at $\simeq 0.55\,M_{\odot}$ rather than the canonical $\simeq 0.60\,M_{\odot}$ value (Blouin and Dufour 2019; Coutu et al. 2019; Koester and Kepler 2019; Caron et al. 2023).

There also exists a second, more exotic kind of DQ-type white dwarfs, which differ from their classical counterparts in several ways. For reasons that will become obvious, these objects are often called the massive DQ, warm DQ, or hot DQ stars.^5^ From a purely atmospheric point of view, they are both hotter ($25{,}000\,\mathrm{K} \gtrsim T_{\mathrm{eff}} \gtrsim 10{,}000$ K) and more carbon-rich ($\log N_{\mathrm{C}}/N_{ \mathrm{He}} \gtrsim -3.0$). This can be seen in Fig. 5, where they form a second sequence, clearly separate from that of the classical DQ stars (Dufour et al. 2007b, 2008; Coutu et al. 2019; Koester and Kepler 2019). In fact, the surface of most objects either is or could be dominated by carbon rather than helium (and interestingly, traces of hydrogen are often present as well; Koester et al. 2020, Kilic et al. 2024). In addition, they are much more massive ($0.90\,M_{\odot} \lesssim M \lesssim 1.20\,M_{\odot}$; Coutu et al. 2019, Koester and Kepler 2019) and tend to have unusually high space velocities (Cheng et al. 2019; Coutu et al. 2019; Kawka et al. 2023), rotation rates (Williams et al. 2016; Macfarlane et al. 2017), and magnetic fields (Dufour et al. 2013; Williams et al. 2013). Taken together, these characteristics suggest that massive DQ white dwarfs are the products of white dwarf mergers rather than standard single-star evolution (Dunlap and Clemens 2015; Kawka et al. 2023).

Finally, a third category of carbon-polluted white dwarfs, although more marginal, deserves to be mentioned. A few relatively hot DB stars showing a seemingly pure-helium atmosphere in the optical reveal traces of carbon (and nothing else) in the ultraviolet. These objects cluster around $T_{\mathrm{eff}} \simeq 25{,}000$ K and $\log N_{\mathrm{C}}/N_{\mathrm{He}} \simeq -5.5$ in Fig. 5 (Provencal et al. 2000; Petitclerc et al. 2005; Dufour et al. 2010a; Koester et al. 2014b). Only a handful of them are currently known due to the limited availability of ultraviolet spectra. Unlike the hot DQ white dwarfs, they appear to have typical stellar properties, including masses near $\simeq 0.60\,M_{\odot}$ (Koester et al. 2014b). As an aside, note that all three classes of carbon-polluted white dwarfs belong to the wider family of hydrogen-deficient white dwarfs. In contrast, carbon contamination of hydrogen-dominated atmospheres is an extremely rare phenomenon (Liebert 1983; Hollands et al. 2020; Kilic et al. 2024).

Let us now move on to the case of other heavy elements, which is completely different from that of carbon. Metal pollution affects both hydrogen-rich and helium-rich white dwarfs over the entire cooling sequence, giving rise to an array of spectral types including DAZ, DBZ, DOZ, and DZ. Although this phenomenon is frequently detected in the optical through weak lines of one or two elements, it produces a much stronger signature in the ultraviolet, where many additional species can be identified and measured. Consequently, most of what we know about the metal abundance pattern of white dwarfs comes from detailed analyses of ultraviolet spectra of a relatively small number of objects. For reasons that will appear in due course, we will discuss white dwarfs hotter and cooler than $T_{\mathrm{eff}} \simeq 30{,}000$ K separately.

At $T_{\mathrm{eff}} \gtrsim 30{,}000$ K, the detectability of heavy elements in the optical is particularly poor, hence metal lines are usually seen exclusively in the ultraviolet. Despite this observational challenge, an astonishing number of elements have been identified at the surface of hot white dwarfs. The most common species are carbon, nitrogen, oxygen, silicon, phosphorus, sulfur, iron, and nickel (Holberg et al. 1993; Werner and Dreizler 1994; Dreizler and Werner 1996; Dreizler 1999; Barstow et al. 2003; Good et al. 2005; Vennes et al. 2006; Preval et al. 2013; Barstow et al. 2014; Werner et al. 2017; Preval et al. 2019), but the full list of detected elements also includes aluminium, argon, chromium, manganese, cobalt, copper, zinc, gallium, germanium, arsenic, selenium, bromine, krypton, strontium, zirconium, molybdenum, indium, tin, antimony, tellurium, iodine, xenon, cesium, and barium (Chayer et al. 2005; Vennes et al. 2005; Werner et al. 2007, 2012; Rauch et al. 2013, 2014a,b, 2015a,b, 2016a,b; Hoyer et al. 2017; Rauch et al. 2017a,b; Hoyer et al. 2018; Werner et al. 2018a,b; Löbling et al. 2020; Rauch et al. 2020; Chayer et al. 2023). The measured abundances span several orders of magnitude and can vary significantly from one object to another, even when the atmospheric parameters are otherwise similar. Despite this large dispersion, a few global trends stand out. First, the incidence of heavy-element pollution declines along the cooling sequence, from 60–70% at $T_{\mathrm{eff}} \gtrsim 60{,}000$ K to 20–30% at $T_{\mathrm{eff}} \simeq 30{,}000$ K (Barstow et al. 2014). The level of contamination seems to follow an analogous but weaker correlation, with cooler objects generally having slightly lower abundances. Furthermore, at a given temperature, helium-dominated white dwarfs tend to be more polluted than their hydrogen-dominated counterparts (Dreizler and Werner 1996; Dreizler 1999; Barstow et al. 2003; Good et al. 2005; Vennes et al. 2006; Barstow et al. 2014; Werner et al. 2018b; Rauch et al. 2020).

Although metals usually produce spectral features only in the ultraviolet, they can manifest themselves indirectly in the optical through their influence on the hydrogen and helium lines. In particular, several hot DA white dwarfs are afflicted by the so-called Balmer-line problem, wherein the core of the H$\alpha $ and H$\beta $ lines is poorly reproduced by atmosphere models assuming a pure-hydrogen composition (Napiwotzki 1992; Bergeron et al. 1994; Napiwotzki and Rauch 1994; Napiwotzki 1999; Gianninas et al. 2010). A similar issue affects the ionised helium features of hot DO white dwarfs (Dreizler and Werner 1996; Reindl et al. 2014a; Werner et al. 2014; Bédard et al. 2020; Reindl et al. 2023). The Balmer-line problem was shown to vanish when heavy elements are properly included in the atmosphere models, as they alter the temperature stratification of the outer layers, which in turn impacts the shape of the spectral features (Bergeron et al. 1993; Werner 1996a; Gianninas et al. 2010). In accordance with ultraviolet studies of metal pollution, the incidence of the Balmer-line problem quickly decreases along the cooling sequence, such that most affected objects have $T_{\mathrm{eff}} \gtrsim 60{,}000$ K (Gianninas et al. 2010; Bédard et al. 2020). Interestingly, the phenomenon is more common and more severe in (homogeneous) DAO stars than in DA stars, indicating that the helium and metal contents are correlated and thus due to the same physical mechanism (Bergeron et al. 1994; Napiwotzki 1999; Good et al. 2005; Gianninas et al. 2010; Bédard et al. 2020; Reindl et al. 2023).

At $T_{\mathrm{eff}} \lesssim 30{,}000$ K, the fraction of white dwarfs exhibiting traces of heavy elements remains roughly constant at 20–30% along the rest of the cooling sequence (Zuckerman and Reid 1998; Zuckerman et al. 2003, 2010; Koester et al. 2014a; Manser et al. 2024; O’Brien et al. 2024). Because the visibility of metals increases as the temperature decreases, optical lines become more common in this range, especially those of calcium. It is therefore possible to estimate surface calcium abundances for large samples of DAZ, DBZ, and DZ stars. The measured abundances roughly span $-10.0 \lesssim \log N_{\mathrm{Ca}}/N_{\mathrm{H}} \lesssim -6.0$ for hydrogen-atmosphere white dwarfs and $-12.0 \lesssim \log N_{\mathrm{Ca}}/N_{\mathrm{He}} \lesssim -6.0$ for helium-atmosphere white dwarfs (Zuckerman and Reid 1998; Zuckerman et al. 2003; Koester et al. 2005; Dufour et al. 2007a; Zuckerman et al. 2010; Koester et al. 2011; Koester and Kepler 2015; Hollands et al. 2017; Coutu et al. 2019; Blouin and Xu 2022; Badenas-Agusti et al. 2024). The difference is simply due to the higher transparency of a helium plasma compared to a hydrogen plasma at the same temperature, which enables the detection of smaller amounts of contaminants. Moreover, in both cases, the lower limits are attainable only at $T_{\mathrm{eff}} \lesssim 10{,}000$ K, where the transparency of the atmosphere is highest. Worthy of mention is the fact that DBZ and DZ white dwarfs are more common than their DAZ counterparts, a disparity that can be explained by the above-mentioned visibility effect (Koester et al. 2005; McCleery et al. 2020; Blouin and Xu 2022; Caron et al. 2023; O’Brien et al. 2024). Another interesting trend specifically concerns the DB population, among which the presence of calcium is correlated with the presence of hydrogen; in other words, several (classical) DBA stars are in fact DBAZ stars (Koester et al. 2005; Koester and Kepler 2015; Gentile Fusillo et al. 2017). This suggests that the trace hydrogen and metals observed in helium-atmosphere white dwarfs may have a common origin.

As in hotter white dwarfs, blue or ultraviolet spectroscopy allows the detection of many additional species, most notably oxygen, sodium, magnesium, aluminium, silicon, titanium, chromium, manganese, iron, and nickel. This list includes all the major rock-forming elements. In fact, and quite interestingly, a plethora of studies have revealed that the metal abundance pattern of cool white dwarfs typically roughly resembles that of the Earth’s interior (Zuckerman et al. 2007; Dufour et al. 2010b; Klein et al. 2010; Farihi et al. 2011; Klein et al. 2011; Melis et al. 2011; Zuckerman et al. 2011; Dufour et al. 2012; Gänsicke et al. 2012; Jura et al. 2012; Farihi et al. 2013; Xu et al. 2013; Jura and Young 2014; Koester et al. 2014a; Xu et al. 2014; Raddi et al. 2015; Wilson et al. 2015; Farihi 2016; Farihi et al. 2016; Gentile Fusillo et al. 2017; Xu et al. 2017; Hollands et al. 2018a; Blouin et al. 2019a; Swan et al. 2019; Xu et al. 2019; Fortin-Archambault et al. 2020; Hoskin et al. 2020; Hollands et al. 2021; Izquierdo et al. 2021; Kaiser et al. 2021; Klein et al. 2021; Elms et al. 2022; Hollands et al. 2022; Johnson et al. 2022; Doyle et al. 2023; Swan et al. 2023; Rogers et al. 2024; Vennes et al. 2024). Besides, note that calcium and magnesium are frequently observed in cool white dwarfs but never in their hotter siblings discussed above, which indicates a fundamental difference in the source of heavy-element pollution.

### The bifurcation in the Gaia colour–magnitude diagram

**Fig. 6:**
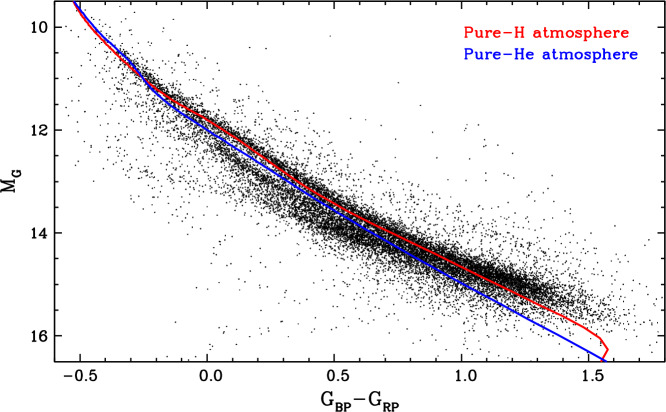
Gaia colour–magnitude diagram of white dwarfs located within 100 pc of the Sun (black dots, taken from the Montreal White Dwarf Database; Dufour et al. 2017). Only the objects with a parallax error smaller than 10% are displayed. Also shown are theoretical evolutionary sequences for pure-hydrogen atmosphere (red curve) and pure-helium atmosphere (blue curve) white dwarfs with a stellar mass of 0.60 $M_{\odot}$, calculated using the atmosphere models of Bergeron et al. (2011), Tremblay et al. (2011), and Blouin et al. (2019b)

Spectroscopy is essential to unravel the nature and abundances of the trace elements present at the surface of individual white dwarfs. However, as it turns out, photometry can also provide insights on the overall atmospheric contamination of large samples of objects. A now famous example of this is the bifurcation of the white dwarf sequence into two distinct branches, denoted A and B, in the Gaia colour–magnitude diagram, reproduced here in Fig. 6 (Gaia Collaboration et al. 2018a). The A and B branches were immediately interpreted as the cooling tracks of hydrogen-dominated and helium-dominated white dwarfs, respectively, offset in colour and magnitude due to the different atmospheric opacities. One one hand, pure-hydrogen atmosphere models indeed predict that a typical 0.60 $M_{\odot}$ white dwarf should evolve along the A branch. On the other hand, it was found that the analogous pure-helium atmosphere model sequence does not coincide with the B branch and instead falls in between the two branches, where very few stars are observed (Jiménez-Esteban et al. 2018; Kilic et al. 2018; Bergeron et al. 2019; Gentile Fusillo et al. 2019).

The bifurcation appears at relatively low effective temperatures, $11{,}000\,\mathrm{K} \gtrsim T_{\mathrm{eff}} \gtrsim 7000$ K, and the subsample of white dwarfs with SDSS spectroscopy confirms that the lower branch consists mostly of helium-rich objects with spectral types DC, DQ, and DZ (Bergeron et al. 2019; Gentile Fusillo et al. 2019, 2020). The location of these stars below the theoretical 0.60 $M_{\odot}$ pure-helium cooling track suggests that they have either higher masses (making them smaller and thus less luminous) or atmospheric contaminants providing an additional source of opacity (Jiménez-Esteban et al. 2018; Kilic et al. 2018; Bergeron et al. 2019; Gentile Fusillo et al. 2019; Ourique et al. 2019, 2020). The first hypothesis can be rejected on the grounds that the direct precursors of B-branch white dwarfs, the slightly hotter DB(A)-type stars, almost all have typical masses near 0.60 $M_{\odot}$. On the other hand, the second explanation is supported by the observation that part of the B-branch population is made up of DQ and DZ stars, which obviously do not have a pure-helium surface. And indeed, it is well-known that carbon and other metals not only produce spectral features, but also affect the overall energy distribution and thus the colours of cool helium-rich white dwarfs (Dufour et al. 2005, 2007a; Caron et al. 2023). However, this does not completely solve the problem, as DQ and DZ stars collectively account for only 40–50% of the cool helium-dominated population, while the bulk of the remainder consists of featureless DC white dwarfs seemingly devoid of contaminants (Bergeron et al. 2019; McCleery et al. 2020; Caron et al. 2023; O’Brien et al. 2024).

The key realisation was that some elements may be present in amounts too low to produce optical lines but still high enough to alter the energy distribution. This is the case of hydrogen, which becomes very difficult to detect spectroscopically at $T_{\mathrm{eff}} \lesssim 10{,}000$ K (as shown in Fig. 4) but still influences the continuum opacity of the atmosphere. It was demonstrated that a surface abundance of $\log N_{\mathrm{H}}/N_{\mathrm{He}} \simeq -5.0$ is sufficient to bring the helium-atmosphere model sequence into agreement with the observed B branch (Bergeron et al. 2019; Gentile Fusillo et al. 2020; Kilic et al. 2020; Gentile Fusillo et al. 2021). Moreover, carbon was shown to have such an effect as well, with surface abundances ≃ 1–2 dex lower than measured in classical DQ stars being sufficient in this case (Blouin et al. 2023a; Camisassa et al. 2023). Therefore, the bifurcation hints that the majority of cool DC white dwarfs have undetected traces of hydrogen and/or carbon in their atmosphere.^6^ However, the true amounts of hydrogen and/or carbon cannot be determined using Gaia data alone, as the effects of these elements are degenerate. Fortunately, we will see later that theoretical modelling of the transport of hydrogen and carbon in helium-rich white dwarfs sheds considerable light on this question.

## Theoretical interpretation

The first part of this review was devoted to the description of the numerous pieces of empirical evidence for spectral evolution. This multifaceted picture is the net observable result of an array of physical mechanisms that modify the distribution of chemical elements in the envelope of cooling white dwarfs. In this second part, we approach the subject from a theoretical angle: given a plausible initial composition, what do current models of element transport predict, and how well do these predictions match the empirical evidence? We ultimately seek a unified theory of spectral evolution, constraining the importance of the relevant physical processes and explaining all the observed trends at once. This field of research relies on detailed numerical simulations where both white dwarf cooling and element transport are considered simultaneously and self-consistently, which can be particularly challenging given the extremely wide range of characteristic time and space scales involved. We will first review the fundamentals of chemical transport in stellar envelopes, and then describe in detail how the chemical structure of white dwarfs is expected to change along the cooling sequence according to state-of-the-art simulations. As the chain of events depends on the initial composition, our discussion will be divided into three sections corresponding to three different types of white dwarf progenitors.

### Preliminaries: element transport mechanisms

The transport of chemical elements in stars is a rich and complex topic on which there are many comprehensive reviews, such as those of Michaud et al. (2015) and Salaris and Cassisi (2017). Here, we only give a brief overview of the transport mechanisms thought to be important in white dwarf envelopes: atomic diffusion, convection, radiative levitation, radiative winds, and external accretion. These transport mechanisms can be divided into two categories: diffusion and levitation are microscopic processes, acting on individual chemical species differentially, while convection, winds, and accretion are macroscopic processes, impacting the stellar plasma as a whole.

Generally speaking, **diffusion** denotes the net movement of the chemical constituents of a fluid arising from interparticle collisions in the presence of a spatial gradient in some physical quantity. In stars, the three main types of diffusive transport are chemical diffusion, gravitational settling, and thermal diffusion, which are induced by composition, pressure, and temperature gradients, respectively. On one hand, gravitational settling and thermal diffusion cause heavier particles to move towards regions of higher pressure and temperature, that is, towards the centre of the star. These mechanisms thus tend to sort the elements vertically according their atomic weight, with lighter ones floating above heavier ones. On the other hand, chemical diffusion tends to oppose this separation, as it is directed against the composition gradient and thereby mixes the different species. In the specific case of white dwarfs, gravitational settling is by far the dominant type of diffusive transport. At the surface, the time scale for this process is of the order of a year, much shorter than the time scale for the cooling of the star (Paquette et al. 1986; Dupuis et al. 1992; Koester and Wilken 2006; Koester 2009; Bauer and Bildsten 2019; Heinonen et al. 2020). This means that complete element separation is achieved practically instantaneously in the absence of competing mechanisms, as we have already mentioned a few times. However, it is important to note that the diffusion time scale increases with depth and reaches hundreds of millions of years at the bottom of the envelope, such that the composition there remains unaltered for a significant portion of the cooling process (Fontaine and Michaud 1979; Dehner and Kawaler 1995). Thermal diffusion and chemical diffusion also occur in white dwarfs but have minor effects. Thermal diffusion slightly accelerates element separation in the envelope of hot white dwarfs but becomes negligible as the temperature decreases (Paquette et al. 1986; Althaus and Córsico 2004). Chemical diffusion induces mixing at the boundary between regions of different compositions, resulting in narrow but continuous transition zones (Arcoragi and Fontaine 1980; Vennes et al. 1988).

**Radiative levitation** is the name given to selective chemical transport arising from interactions between matter and radiation. As they propagate throughout the envelope, photons can transfer part of their net outward momentum to highly absorbing atoms, which are thereby supported against gravitational settling. The efficiency of this support is determined by the radiative force, which itself depends on the flux of radiation and the opacity of a given element. Consequently, this transport mechanism mainly affects metals (which are very opaque in the ultraviolet) in luminous stars (which have strong radiation fields). This is also true for white dwarfs: theoretical calculations show that radiative levitation can indeed maintain small amounts of heavy elements at the surface of hot, luminous white dwarfs (Chayer et al. 1995a,b; Dreizler and Wolff 1999; Dreizler 1999; Schuh et al. 2002; Rauch et al. 2013; Chayer 2014; Koester et al. 2014a). Although the magnitude and behaviour of the contamination are specific to each chemical species, the predicted abundances are typically highest at the beginning of the cooling sequence and decrease with decreasing effective temperature. Furthermore, the amount of levitating elements is expected to be higher in helium-rich white dwarfs than in their hydrogen-rich counterparts, mainly because of the higher metal-line opacity in a helium-dominated plasma. The cooling of the star eventually makes radiative levitation completely inefficient; this cutoff occurs at $T_{\mathrm{eff}} \simeq 20{,}000$–40,000 K depending on the element (Chayer et al. 1995a,b). We note that existing calculations assume a strict equilibrium between gravitational and radiative forces, which results in a unique abundance pattern for a given set of atmospheric parameters.

**Fig. 7:**
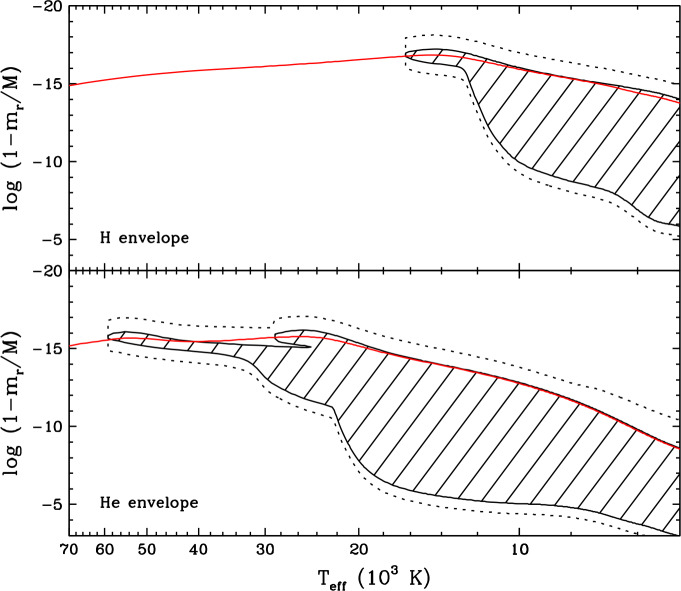
Vertical extent of the convection zone (hatched area) as a function of effective temperature in hydrogen-rich (top panel) and helium-rich (bottom panel) white dwarf envelope models. The position in the star is measured in terms of the fraction of the total mass located outside a given radius, such that the surface is towards the top and the core is towards the bottom. These models were computed with the STELUM code (Bédard et al. 2022b) assuming a stellar mass of 0.60 $M_{\odot}$ and the ML2 version of the mixing-length theory (Tassoul et al. 1990). In each panel, the red line shows the position of the photosphere. As a very rough indication of the expected extent of convective overshoot, the dotted lines denote two pressure scale heights above and below the formally convective region

Let us now turn to macroscopic transport, starting with **convection**, arguably one of the most important physical mechanisms in stellar astrophysics. In stellar envelopes, convection can be described as a large-scale, cyclic flow of matter whereby hot fluid ascends while cool fluid descends, hence causing very efficient heat transport towards the surface. This process arises in regions where heat transport through radiation is ineffective, for instance as a result of a large overall opacity. The rapid fluid motions also generate element transport, more specifically by thoroughly mixing the various chemical species present within the convective region. In other words, convection completely wipes out the segregation effect of diffusion and produces a perfectly flat composition profile. In a white dwarf, the envelope develops a convection zone as the temperature decreases and the main chemical constituent recombines and thus becomes very opaque. For most applications, convection is modelled using the so-called Schwarzschild criterion and mixing-length theory, a one-dimensional approximation of the inherently three-dimensional process (Fontaine and van Horn 1976; Tassoul et al. 1990; MacDonald and Vennes 1991; Benvenuto and Althaus 1997; Althaus and Benvenuto 1998; Koester 2009; Rolland et al. 2018; Bauer and Bildsten 2019; Bédard et al. 2022b). Figure 7 shows the predicted vertical extent of the convective region in hydrogen-rich (top panel) and helium-rich (bottom panel) white dwarf envelope models as a function of effective temperature. The position in the star is measured in terms of the mass $m_{r}$ located inside radius $r$, such that $1-m_{r}/M$ represents the fraction of the total mass located outside radius $r$. In a hydrogen-rich white dwarf, the convection zone appears at $T_{\mathrm{eff}} \simeq 16{,}000$ K but is initially limited to the atmospheric layers. It then starts to expand significantly at $T_{\mathrm{eff}} \simeq 12{,}000$ K and ends up spanning a large portion of the envelope. An analogous behaviour is seen in a helium-rich white dwarf but at a higher temperature: the convection zone appears at $T_{\mathrm{eff}} \simeq 60{,}000$ K and expands downward at $T_{\mathrm{eff}} \simeq 20{,}000$ K.

The mixing-length theory provides a very coarse representation of convection, where the fluid motions abruptly stop at the top and bottom boundaries of the convective region. In reality, the convective flows are expected to overshoot beyond the boundaries, thereby inducing chemical mixing over a larger region. In one-dimensional white dwarf models such as those displayed in Fig. 7, the extent of convective overshoot is essentially treated as a free parameter and thus introduces significant uncertainty (Paxton et al. 2011; Rolland et al. 2018; Koester et al. 2020; Bédard et al. 2022b). In the last decade or so, a more accurate description of convection in white dwarfs has become possible thanks to elaborate three-dimensional radiation-hydrodynamics simulations (Tremblay et al. 2013; Cukanovaite et al. 2018; Kupka et al. 2018; Cunningham et al. 2019). These are too demanding to be coupled to white dwarf cooling calculations, but can be used to calibrate the mixing-length theory (Tremblay et al. 2015; Cukanovaite et al. 2019) and to estimate the extent of convective overshoot (Kupka et al. 2018; Cunningham et al. 2019). The latter works reveal that overshoot can generate chemical mixing up to a distance of a few pressure scale heights beyond the convection zone. For illustrative purposes, the dotted lines in Fig. 7 indicates the depths corresponding to two pressure scale heights above and below the formally convective region. However, such hydrodynamics-based constraints have so far been obtained only for relatively hot white dwarfs with shallow convection zones ($\log (1-m_{r}/M) \lesssim -12.0$), and therefore our understanding of convective overshoot remains largely incomplete. Besides, another convection-like process that can generate chemical mixing in formally non-convective regions is the so-called thermohaline or fingering instability. This instability arises in the presence of an inverted composition gradient, where heavier material sits on top of a lighter fluid, and induces a slow homogenisation of the different chemical layers. One-dimensional models predict that thermohaline mixing should occur in the envelope of accreting (see below) hydrogen-rich white dwarfs (Deal et al. 2013; Wachlin et al. 2017; Bauer and Bildsten 2018, 2019; Wachlin et al. 2022; Dwomoh and Bauer 2023), although these results have been disputed (Koester 2015).

**Radiative winds** constitute another transport mechanism believed to be important in the context of spectral evolution, especially at the beginning of the cooling sequence. These winds are essentially an extreme, macroscopic version of radiative levitation: the outward radiative force due to metal absorption exceeds that of gravity, such that hydrostatic equilibrium becomes impossible. Consequently, the outer layers of the star experience an outward radial motion and are successively peeled off, resulting in continuous mass loss. This outflow imparts the same velocity to all chemical species present in the envelope, thereby preventing element separation through diffusion, similarly to convection. The quantity typically employed to measure the strength of a stellar wind is the rate of mass loss. For radiation-driven winds, the mass-loss rate is determined primarily by the stellar luminosity, and secondarily by the heavy-element content; the wind is stronger in a more luminous and/or more metal-rich star (Kudritzki and Puls 2000). The question of whether hot white dwarfs can power such winds in spite of their high gravity has received very little theoretical attention, but the few available calculations indicate they indeed can at the very beginning of the cooling sequence (Unglaub and Bues 2000; Unglaub 2008). The predicted mass-loss rates, $\simeq 10^{-12}$–$10^{-11}\,M_{\odot}\,\mathrm{yr}^{-1}$, are too low to produce a spectroscopic signature but sufficiently high to considerably slow down gravitational settling in the outer layers. Due to the general lack of wind calculations, models of chemical transport in hot white dwarfs must rely on simplistic analytical expressions for the mass-loss rate, usually considering only the luminosity dependence and calibrated on previous phases of stellar evolution (Unglaub and Bues 1998, 2000; Quirion et al. 2012; Bédard et al. 2022b).

Finally, the external **accretion** of matter can alter the surface composition of white dwarfs. This process can not only enhance the abundance of elements that are already there, but also, unlike the other transport mechanisms, add new species to the chemical reservoir of the star. Possible sources of accretion material are the interstellar medium (Alcock and Illarionov 1980; MacDonald and Vennes 1991; Dupuis et al. 1993a) and the immediate circumstellar environment, including a stellar or substellar companion (Sion 1999; Debes and Sigurdsson 2002; Veras 2021). In white dwarfs, it is latter source that turns out to be prevalent, as we will see below.

### Hydrogen-rich progenitor

The chemical evolution of a given white dwarf is largely dictated by the initial condition, that is, by the envelope composition of its immediate precursor. This initial reservoir not only determines the nature of the elements that will potentially appear at the surface, but also impacts the efficiency of transport mechanisms, most notably convection, along the cooling sequence. We will begin by considering the most common and straightforward scenario, arising from a standard hydrogen-rich progenitor. According to stellar evolution models, the majority of stars should retain a relatively thick hydrogen layer of $\simeq 10^{-4}\,M$ as they become white dwarfs (Iben and Tutukov 1984; Renedo et al. 2010; Romero et al. 2012). This necessarily implies that the surface should remain dominated by hydrogen along the entire cooling sequence. Indeed, among the transport mechanisms discussed in Sect. 3.1, only convection is powerful enough to change the bulk atmospheric composition altogether. As shown in Fig. 7, the convection zone present in the hydrogen layer at $T_{\mathrm{eff}} \lesssim 16{,}000$ K is always limited to the outer $\simeq 10^{-5}\,M$ and thus never reaches the underlying helium mantle. Therefore, the hydrogen and helium layers never mix, implying that the hydrogen-rich nature of the atmosphere cannot be altered. This canonical scenario naturally explains the first of the three main evolutionary channels identified in Sect. 2.2, namely, that involving enduring DA stars and representing about 75% of the white dwarf population (Tremblay and Bergeron 2008; Rolland et al. 2018; Bédard et al. 2020; Cunningham et al. 2020).

Of course, transport processes other than convection can still affect the surface chemistry by acting as sources of minor contaminants. At the hot end of the cooling sequence, the presence of helium and heavier elements in most hydrogen-dominated white dwarfs indicates that the primordial solar mixture has not yet been fully purified by gravitational settling. At first glance, two transport mechanisms appear as plausible candidates to explain this observation: radiative levitation and radiative winds. In both scenarios, it is the strong outward radiation field that prevents the sedimentation of trace elements, and the question essentially boils down to whether it does so in or out of hydrostatic equilibrium. Given the high surface gravity of white dwarfs, the occurrence of a full-blown stellar wind historically appeared unlikely, and thus radiative levitation was initially considered as the most sensible hypothesis. However, theoretical calculations demonstrated that radiative levitation alone is glaringly insufficient to account for the amount of helium observed in hot DAO stars (Vennes et al. 1988; MacDonald and Vennes 1991). Furthermore, the assumption of a strict equilibrium between gravitational and radiative forces leads to the prediction of a unique surface composition for a given set of atmospheric parameters, which is at odds with the large spread in measured heavy-element abundances (Chayer et al. 1995a,b; Barstow et al. 2003, 2014).

This leaves a radiative wind as the only viable explanation for the helium contamination of hot DAO white dwarfs. Although the theoretical basis is admittedly uncertain, the wind scenario is fully consistent not only with the inferred helium abundances (Unglaub and Bues 1998, 2000), but also with several other properties of the hot DAO population. First, these objects are observed to have a chemically homogeneous atmosphere, as expected in the presence of a wind. Second, the strength of a radiative wind is mainly determined by the surface luminosity, with more luminous stars generating stronger winds. This naturally explains why helium contamination becomes both less frequent (as measured by the overall fraction of DAO stars) and less significant (as measured by abundances of individual objects) along the cooling sequence. This may also explain why hot DAO white dwarfs tend to have low masses (corresponding to large radii and thus high luminosities). Third, as the wind is driven by metals, we necessarily expect a correlation between the presence of helium and heavier elements, which is indeed observed through severe forms of the Balmer-line problem in many hot DAO stars (Bergeron et al. 1994; Napiwotzki 1999; Gianninas et al. 2010; Bédard et al. 2020).

The exact effective temperature at which radiative winds vanish is unknown and likely varies from one object to another depending on the stellar mass and the metal content. However, we can conservatively assert that such winds cannot exist at $T_{\mathrm{eff}} \lesssim 60{,}000$ K (Unglaub and Bues 2000; Unglaub 2008). This crude theoretical limit is in line with the disappearance of hot homogeneous DAO stars, which then turn into normal DA stars. Nevertheless, a significant fraction of these cooler hydrogen-rich white dwarfs still show traces of heavy elements, indicating that other transport mechanisms take over in supporting or replenishing the atmospheric metal content. Radiative levitation may play a role, as the observed decrease in pollution incidence along the cooling sequence is roughly consistent with the expected decline in radiative support (Chayer et al. 1995a,b; Barstow et al. 2003, 2014). However, this process alone still fails to account for the large object-to-object variations in measured abundances and for all instances of metal pollution below $T_{\mathrm{eff}} \simeq 20{,}000$ K (at best). Therefore, the only possible interpretation is that heavy elements are currently being accreted (or have very recently been accreted) at the surface of these white dwarfs. Historically, the source of this external material was believed to be the interstellar medium (Dupuis et al. 1993a,b; Zuckerman et al. 2003; Koester et al. 2005; Koester and Wilken 2006). It is now well established that white dwarfs actually accrete the remains of their planetary systems: the orbiting planets and asteroids are disrupted by the strong tidal forces and subsequently fall onto the stellar surface (Zuckerman et al. 2007; Farihi et al. 2010; Barstow et al. 2014; Jura and Young 2014; Koester et al. 2014a; Farihi 2016; Schreiber et al. 2019; Veras 2021). This naturally explains why the metal abundance pattern of many cool white dwarfs is consistent to first order with the bulk composition of the Earth. The accretion scenario also provides the element of randomness that characterises the occurrence and magnitude of metal pollution.

The accretion of planetary debris onto white dwarfs provides a unique way to probe the internal structure of exoplanets. Nevertheless, the abundances measured in the atmosphere of the white dwarf do not strictly reflect the original composition of the accreted body, because the material deposited at the surface is affected by the transport mechanisms at work in the envelope. On one hand, the accreted matter sinks into the star due to gravitational settling, and this process unfolds at different rates for different elements. On the other hand, the sinking material can be slowed down by radiative levitation (in sufficiently hot white dwarfs), by mixing within a convection zone, and possibly by mixing arising from the thermohaline instability. By modelling this interplay between accretion, diffusion, and mixing (Dupuis et al. 1993a; Koester 2009; Bauer and Bildsten 2019; Wachlin et al. 2022), it is possible to trace back the mass and bulk composition of the accreted asteroid or planet (see the references given in the last paragraph of Sect. 2.4). In some cases, it may even be possible to constrain the original core, mantle, and crust stratification (Jura and Young 2014; Harrison et al. 2018; Bonsor et al. 2020; Harrison et al. 2021; Buchan et al. 2022; Swan et al. 2023).

### Helium-rich progenitor: the effect of residual hydrogen

As we have seen in Sect. 2.2, about 25% of all stars enter the cooling sequence with a strongly hydrogen-deficient atmosphere. These objects have apparently lost the greater part of their residual hydrogen layer, implying a non-standard evolutionary history. The main such evolutionary pathway is thought to be the so-called born-again scenario, where a soon-to-be white dwarf experiences a late helium-shell flash and thus rapidly grows back to giant dimensions. This violent event triggers extensive convection in the envelope, such that the hydrogen is deeply engulfed into the star and thereby almost completely burned, while some carbon and oxygen are dredged up to the surface. When nuclear burning ceases and the star contracts again to become a white dwarf (for good this time), the outcome is an envelope made of helium, carbon, and oxygen in similar proportions (Iben et al. 1983; Herwig et al. 1999; Blöcker 2001; Althaus et al. 2005b; Lawlor and MacDonald 2006; Miller Bertolami et al. 2006; Miller Bertolami and Althaus 2006; Werner and Herwig 2006). This scenario is strongly supported by observations, as most hydrogen-deficient pre-white dwarfs indeed exhibit a hot helium–carbon–oxygen atmosphere, corresponding to the PG 1159 spectral class^7^ (Werner et al. 1991; Dreizler and Heber 1998; Hügelmeyer et al. 2005, 2006; Werner and Herwig 2006; Werner et al. 2014; Werner and Rauch 2014; Reindl et al. 2023). A few objects known as hybrid PG 1159 stars also show traces of hydrogen, indicating that they did not get rid of their whole hydrogen content (Werner and Herwig 2006; Werner et al. 2014). The measured abundances are relatively large ($\log N_{\mathrm{H}}/N_{\mathrm{He}} \simeq 0.0$), but given the difficulty of detecting hydrogen in a hot helium-dominated atmosphere, lower amounts of hydrogen may be present in most born-again stars (Althaus et al. 2005b; Lawlor and MacDonald 2006; Miller Bertolami et al. 2006, 2017). In short, the appropriate initial condition to model this spectral evolution channel is a helium-rich envelope containing trace hydrogen as well as significant carbon and oxygen. The initial abundances of these elements are additional parameters that can be varied over plausible ranges, thereby making the predicted behaviours much more complex and diverse than in the case of a standard hydrogen-rich progenitor. This was first demonstrated by Althaus et al. (2005b) thanks to full evolutionary calculations linking the born-again episode to the white dwarf phase. In this section, we will first focus on the effect of residual hydrogen.

Before diving into the topic, we note that the considerations of the previous section regarding the origins of heavy elements in hydrogen-dominated white dwarfs also apply to their helium-dominated counterparts (with the obvious exceptions of carbon and oxygen). The primordial metals are initially supported in the atmosphere by the radiative wind, and then by radiative levitation once the wind has faded. These mechanisms are expected to be more efficient in helium-rich white dwarfs, in line with the observed trend for these stars to have higher metal abundances (Chayer et al. 1995a; Unglaub and Bues 2000). At lower effective temperature, the detected heavy elements originate from the accretion of tidally disrupted asteroids and planets. A key difference for the interpretation of the observed surface abundances is that the convection zone of helium-dominated objects is much deeper at a given temperature (see the bottom panel of Fig. 7). The accreted material is thus mixed within a larger region, but also remains there longer as the settling time scales of metals below the deep convection zone reach millions of years (Paquette et al. 1986; Dupuis et al. 1993a; Koester 2009). The extended convective mixing also greatly diminishes the role of thermohaline mixing compared to hydrogen-rich white dwarfs (Deal et al. 2013; Bauer and Bildsten 2019).

**Fig. 8:**
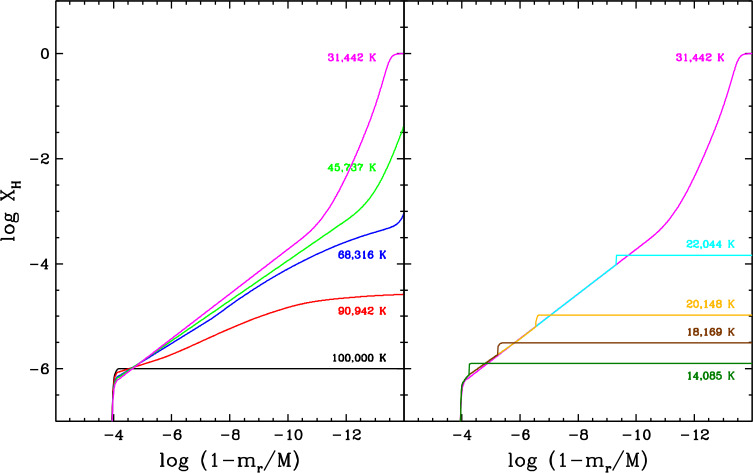
Hydrogen mass fraction profile at various effective temperatures in a typical simulation of element transport in helium-rich white dwarfs. The position in the star is measured in terms of the fraction of the total mass located outside a given radius, such that the surface is towards the right and the core is towards the left. This particular simulation is taken from Bédard et al. (2023), and assumes a stellar mass of 0.60 $M_{\odot}$ and an initial hydrogen mass fraction $X_{\mathrm{H}} = 10^{-6}$. The effective temperature decreases monotonically with time; the left panel illustrates the float-up process at high temperature, while the right panel illustrates the convective dilution process at low temperature

Let us now come back to the main question of this section: what is the expected chemical evolution of a helium-dominated progenitor harbouring residual hydrogen? To aid the discussion, Fig. 8 displays the results of a theoretical simulation of element transport in such an object; more specifically, the predicted hydrogen abundance profile is shown at various stages of the cooling process (Bédard et al. 2023). The position in the star is measured in terms of the quantity $1-m_{r}/M$ introduced earlier in Sect. 3.1. This particular model assumes a relatively low initial hydrogen content: a uniform mass fraction $X_{\mathrm{H}} = 10^{-6}$ in the outer 10$^{-4}\,M$ of the star (corresponding to a total hydrogen mass of 10$^{-10}\,M$). As shown in the left panel of Fig. 8, the hydrogen diluted within the envelope floats towards the surface due to gravitational settling. However, this so-called float-up process is initially slowed down by the radiative wind, as the latter tends to prevent any chemical differentiation in the outer part of the envelope. As the white dwarf cools and the wind accordingly fades, diffusion becomes increasingly efficient and eventually leads to the formation of a thin pure-hydrogen layer at the surface (Unglaub and Bues 2000; Althaus et al. 2005b,a, 2020; Bédard et al. 2022b, 2023). Note, however, that this layer comprises only a small fraction of the total hydrogen content of the white dwarf, the rest of which is still located at great depths where diffusion time scales are much longer (Rolland et al. 2020; Bédard et al. 2023). From an observational point of view, the helium-atmosphere DO star has transformed into a hydrogen-atmosphere DA star. In the simulation shown in Fig. 8, this DO-to-DA transition occurs at a relatively advanced stage, $T_{\mathrm{eff}} \simeq 35{,}000$ K, due to the low initial hydrogen abundance assumed. A progenitor with a higher amount of residual hydrogen develops a hydrogen-rich atmosphere earlier. For instance, the initial condition $X_{\mathrm{H}} = 10^{-3}$ gives rise to a DO-to-DA transition at $T_{\mathrm{eff}} \simeq 70{,}000$ K. Nevertheless, the speed of the float-up process also depends on the strength of the radiative wind, which is poorly constrained and thus introduces significant uncertainty in the predicted transition temperature (Unglaub and Bues 1998, 2000; Bédard et al. 2023).

The newly-formed thin hydrogen layer is short-lived, however, as it is condemned to be wiped out by convection. At this point, two distinct scenarios may take place depending on the exact thickness of the hydrogen shell built by diffusion. On one hand, if the latter extends over less than the outer $\simeq 10^{-14}\,M$ of the star, the underlying helium-rich envelope becomes convective (see the bottom panel of Fig. 7). Overshoot above the convection zone then erodes the hydrogen layer from below and dilutes it within the helium-rich envelope. Observationally, the result is a helium-dominated, hydrogen-contaminated atmosphere, namely, a DBA white dwarf. As the star cools and the convection zone grows deeper, the hydrogen is gradually diluted within an increasingly large region, such that surface hydrogen abundance decreases with time. This process, illustrated in the right panel of Fig. 8, is generally referred to as convective dilution^8^ (MacDonald and Vennes 1991; Rolland et al. 2018, 2020; Bédard et al. 2023). Depending on the thickness of the hydrogen layer, the DA-to-DBA transition is predicted to occur in the range $30{,}000\,\mathrm{K} \gtrsim T_{\mathrm{eff}} \gtrsim 14{,}000$ K, with larger layers giving rise to lower transition temperatures. That said, the exact relation between these two quantities remains significantly uncertain due to our poor knowledge of convective overshoot (Cunningham et al. 2020; Rolland et al. 2020).

On the other hand, a hydrogen shell more massive than $\simeq 10^{-14}\,M$ inhibits the onset of convection in the helium-dominated envelope and thus remains intact a little longer. Nevertheless, the hydrogen layer itself inevitably becomes convective at lower effective temperature (see the top panel of Fig. 7). When the convection zone reaches the underlying helium-rich region, helium is efficiently carried into the outer hydrogen-rich layer; the convection zone accordingly grows deeper, such that more helium is dredged up to the surface, and so on. This runaway process results in a thorough, almost instantaneous mixing of the hydrogen shell within the helium-rich envelope. This phenomenon is known as convective mixing and takes place in the range $14{,}000\,\mathrm{K} \gtrsim T_{\mathrm{eff}} \gtrsim 6000$ K (Baglin and Vauclair 1973; Koester 1976; D’Antona and Mazzitelli 1989; MacDonald and Vennes 1991; Althaus and Benvenuto 1998; Tremblay and Bergeron 2008; Chen and Hansen 2011; Rolland et al. 2018; Cunningham et al. 2020; Bédard et al. 2022b; Bergeron et al. 2022). Similarly to convective dilution, the observational outcome is a white dwarf with a helium-dominated, hydrogen-contaminated atmosphere. However, given the lower effective temperature and the larger hydrogen content, such an object may appear as a helium-rich DA star rather than a DBA star (Chen and Hansen 2011; Rolland et al. 2018; Bédard et al. 2022b).

The theoretical expectations described above can be linked to several observational features detailed in Sect. 2. First, let us recall that while about 25% of all white dwarfs initially have a helium-dominated atmosphere, only about 10% retain this surface composition along the entire cooling sequence. The other 15% experience a helium-to-hydrogen-to-helium transition, as revealed by the V-shaped variation of the helium-rich fraction in the range $75{,}000\,\mathrm{K} \gtrsim T_{\mathrm{eff}} \gtrsim 10{,}000$ K (Fig. 2). This evolutionary channel is naturally explained by the float-up process at high temperature and the convective dilution and mixing mechanisms at low temperature (Fontaine and Wesemael 1987; MacDonald and Vennes 1991; Dreizler and Werner 1996; Bergeron et al. 2011; Rolland et al. 2018; Genest-Beaulieu and Bergeron 2019b; Ourique et al. 2019; Bédard et al. 2020; Cunningham et al. 2020; Rolland et al. 2020; López-Sanjuan et al. 2022; Bédard et al. 2023; Vincent et al. 2024). An interesting corollary is that about 60% of helium-rich progenitors contain sufficient residual hydrogen to undergo spectral evolution, while the rest of them do not. Furthermore, the fact that the variation of the helium-rich fraction is gradual implies that different objects experience their atmospheric transformations at different temperatures and thus possess different hydrogen contents. In principle, the range of initial hydrogen abundances can be estimated by comparing the transition temperatures predicted from transport simulations (Fig. 8) to those “observed” in the white dwarf population (Fig. 2). Such an exercise indicates that the observed spectral evolution arises from helium-rich progenitors with mass fractions in the range $10^{-7} \lesssim X_{\mathrm{H}} \lesssim 10^{-3}$. This rather broad range partially reflects the large uncertainties in the current modelling of radiative winds and convective overshoot, and thus the true range is likely narrower (Bédard et al. 2023).

Furthermore, the transport of hydrogen in helium-rich white dwarfs readily explains the various groups of hybrid-atmosphere objects (with the exception of the very hot homogeneous DAO stars, which we discussed in the last section). In the course of the float-up process, there is inevitably a brief phase where the newly-formed surface hydrogen layer is thin enough that the underlying helium is still visible. Hot white dwarfs exhibiting such a chemically stratified atmosphere can therefore be interpreted as transitional objects in the midst of a DO-to-DA transformation. And indeed, the existence of stratified white dwarfs and the decrease in the helium-rich fraction coincide in terms of temperature range (Figs. 2 and 3), strengthening the idea that these are two manifestations of the same phenomenon (Manseau et al. 2016; Bédard et al. 2020, 2022b, 2023).

**Fig. 9:**
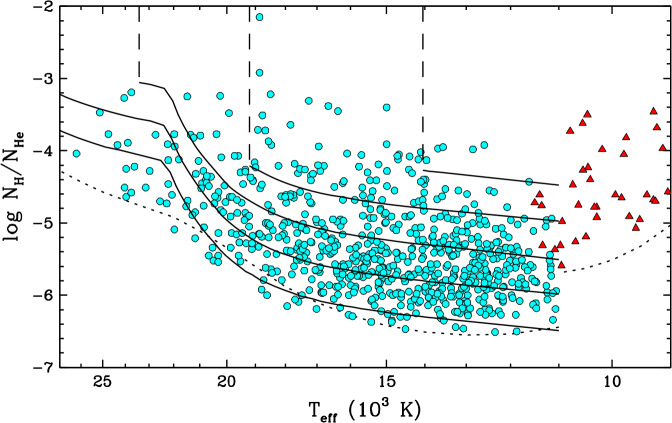
Zoomed-in version of Fig. 4 including theoretical predictions for the convective dilution of hydrogen. These simulations assume a stellar mass of 0.60 $M_{\odot}$ and initial hydrogen mass fractions $X_{\mathrm{H}} = 10^{-7.0}$, $10^{-6.5}$, $10^{-6.0}$, $10^{-5.5}$, and $10^{-5.0}$ (from bottom to top). For each simulation, the transition temperature (dashed part of the curve) is based on the approximate method of Rolland et al. (2020), while the resulting hydrogen abundance (solid part of the curve) is taken from Bédard et al. (2023)

Further along the cooling sequence, the classical DBA stars can be interpreted as products of the convective dilution and mixing mechanisms, as their measured temperatures and compositions are well reproduced by current transport simulations (Rolland et al. 2020; Bédard et al. 2023). This is demonstrated quantitatively in Fig. 9, which is a zoomed-in version of the temperature–abundance diagram where theoretical predictions for various initial conditions were added (the middle curve corresponds to the simulation shown in Fig. 8). We stress once again that our poor knowledge of helium-rich convective overshoot results in significant uncertainties on both the predicted DA-to-DBA transition temperatures (±1000–5000 K; Rolland et al. 2020) and DBA abundances (±0.5 dex; Bédard et al. 2023). For this reason, it would be inadvisable to use the model curves to attempt to infer the total hydrogen content of individual objects. Nevertheless, the overall agreement seen in Fig. 9 strongly supports the view that DBA white dwarfs represent an inevitable stage of spectral evolution. Moreover, we recall that DBA stars account for 60–75% of the DB population, which is consistent with our previous inference that about 60% of helium-rich progenitors go through the DO–DA–DBA channel. Although the theoretical predictions displayed in Fig. 9 are limited to $T_{\mathrm{eff}} > 11{,}000$ K, the atmospheric composition is expected to remain roughly constant at lower temperatures, because the base of the convection zone then barely moves (see the bottom panel of Fig. 7). Therefore, we can deduce from Fig. 9 that DBA white dwarfs are bound to become featureless DC white dwarfs, although those with relatively high hydrogen abundances will first temporarily appear as helium-rich DA stars (Rolland et al. 2018, 2020; Bédard et al. 2023).

For the past three decades or so, the origin of hydrogen in classical DBA white dwarfs has been a topic of considerable debate. Indeed, it has often been proposed that the source of hydrogen is external rather than internal (MacDonald and Vennes 1991; Voss et al. 2007), and more recently that it can be attributed to the accretion of water-rich planets, asteroids, or even comets (Jura and Xu 2010; Klein et al. 2010; Farihi et al. 2011, 2013; Veras et al. 2014; Raddi et al. 2015; Gentile Fusillo et al. 2017; Xu et al. 2017; Hoskin et al. 2020; Izquierdo et al. 2021). This scenario has been preferred by many for two main reasons. First, for a few individual objects, the observed hydrogen and metal abundance pattern indicates ongoing or recent accretion of rocky, water-bearing material (Farihi et al. 2013; Raddi et al. 2015; Gentile Fusillo et al. 2017; Hoskin et al. 2020; Izquierdo et al. 2021). Second, until very recently, simulations of the convective dilution and mixing processes completely failed to reproduce the measured hydrogen abundances of DBA stars, leaving the accretion hypothesis as the only viable alternative (MacDonald and Vennes 1991; Voss et al. 2007; Bergeron et al. 2011; Koester and Kepler 2015; Rolland et al. 2018; Genest-Beaulieu and Bergeron 2019b; Cunningham et al. 2020). The extension of this scenario to the entire DBA population would lead to the drastic implication that 60–75% of all white dwarfs must accrete water-rich bodies within a few 100 Myr on the cooling sequence. Nevertheless, the latest works on convective dilution have identified and corrected a significant shortcoming of previous generations of models (see Bédard et al. 2023 for details), thereby bringing the predictions in excellent agreement with the observations (Fig. 9). Given this improvement, the internal transport of primordial hydrogen now appears as a natural explanation for the existence of the bulk of DBA stars (Rolland et al. 2020; Bédard et al. 2023). However, water accretion must not be totally ruled out: the convective dilution and mixing scenarios predict baseline hydrogen abundances of the right order of magnitude, but it is still plausible that accretion contributes to the observed range. In particular, the model curves of Fig. 9 do not pass through the most hydrogen-rich DBA white dwarfs, which thus require such an external contribution. Finally, we recall that there exists a correlation between hydrogen and metal pollution among the DB population. This suggests that some objects do acquire hydrogen alongside heavier elements through the accretion of planetary debris (Gentile Fusillo et al. 2017).

At $T_{\mathrm{eff}} \lesssim 10{,}000$ K, there is marginal empirical evidence for the occurrence of convective mixing. The possible further increase in the helium-atmosphere fraction (Fig. 2) and the existence of cool helium-rich DA stars with high hydrogen abundances (Fig. 4) suggest that some white dwarfs undergo a late hydrogen-to-helium atmospheric transformation (Fontaine and Wesemael 1987; Bergeron et al. 1997, 2001; Tremblay and Bergeron 2008; Chen and Hansen 2012; Giammichele et al. 2012; Limoges et al. 2015; Rolland et al. 2018; Blouin et al. 2019b; McCleery et al. 2020; Caron et al. 2023; O’Brien et al. 2024). The location of the helium-rich DA stars in the temperature–abundance diagram is well reproduced by simulations of convective mixing (not shown here) assuming relatively thick surface hydrogen layers between $\simeq 10^{-10}\,M$ and 10$^{-8}\,M$ (Chen and Hansen 2011; Rolland et al. 2018; Bédard et al. 2022b; Bergeron et al. 2022). In the bigger picture of spectral evolution, such objects may descend from the most hydrogen-contaminated of the helium-dominated progenitors, namely, the hybrid PG 1159 stars. As they cool further, helium-rich DA white dwarfs are expected to rapidly turn into DC white dwarfs due to the disappearance of hydrogen lines.

**Fig. 10:**
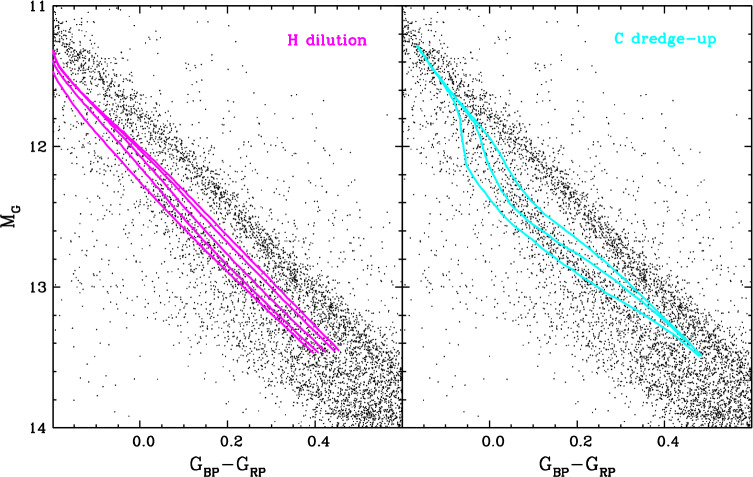
Zoomed-in version of Fig. 6 including theoretical evolutionary sequences for helium-rich white dwarfs contaminated by hydrogen due to convective dilution (left panel) and contaminated by carbon due to convective dredge-up (right panel). These sequences were calculated using the atmosphere models of Blouin et al. (2023a), assuming a stellar mass of 0.60 $M_{\odot}$ and the hydrogen (left panel) and carbon (right panel) abundances predicted by the simulations shown in Figs. 9 and 12, respectively

A key implication of the chemical evolution model outlined in this section is that the majority of cool, helium-atmosphere DC stars should contain spectroscopically undetectable hydrogen. This is especially interesting in the context of the bifurcation in the Gaia colour–magnitude diagram, as the B branch can be explained if DC white dwarfs have $\log N_{\mathrm{H}}/N_{\mathrm{He}} \simeq -5.0$ (Bergeron et al. 2019; Gentile Fusillo et al. 2020; Kilic et al. 2020; Gentile Fusillo et al. 2021). However, this specific requirement proves to be inconsistent with our global understanding of spectral evolution. The direct precursors of most DC stars are DB and DBA stars, for which the surface hydrogen abundance is well constrained. Because the composition of a given DB or DBA white dwarf remains roughly constant as it cools, the DC population should be characterised by a similar range of abundances. The expected trajectory of these objects in the Gaia colour–magnitude diagram is illustrated in the left panel of Fig. 10; the five sequences displayed here assume hydrogen abundances taken from the five simulations of convective dilution shown in Fig. 9. At first glance, the overall agreement with the observed B branch appears satisfactory, but a closer inspection reveals that this is true only for the three lower curves, corresponding to the three most hydrogen-rich models. The two most hydrogen-poor sequences, which are representative of a significant portion of the DBA population (see Fig. 9), fall in between the A and B branches, similarly to the pure-helium atmosphere sequence previously displayed in Fig. 6. In other words, in the descendants of hydrogen-poor DB and DBA stars ($\log N_{\mathrm{H}}/N_{\mathrm{He}} \lesssim -6.0$), the amount of hydrogen is too low to affect the spectral energy distribution. This issue is confirmed by detailed population synthesis calculations assuming a realistic hydrogen abundance distribution, which predict a significant number of objects in between the A and B branches and thus fail to reproduce a clear separation (Blouin et al. 2023a). The conclusion is that the Gaia bifurcation cannot be explained solely by the presence of trace hydrogen in helium-rich white dwarfs.

### Helium-rich progenitor: the effect of residual carbon

As mentioned in the previous section, most hydrogen-deficient PG 1159-type pre-white dwarfs have large amounts of carbon and oxygen in their envelope as a result of their born-again evolution. A range of surface abundances is observed among the PG 1159 population, that is, $0.20 \lesssim X_{\mathrm{C}} \lesssim 0.60$ and $0.02 \lesssim X_{\mathrm{O}} \lesssim 0.20$ in mass fractions (Werner and Herwig 2006; Werner et al. 2014). The purpose of this section is to examine the impact of these elements on the spectral evolution of helium-rich white dwarfs. We will see that oxygen plays a very minor role, while carbon is a critical piece of the puzzle. To alleviate the discussion, we will first ignore the possible presence of trace hydrogen and concentrate on the transport of carbon. We will then come back to the general case where both of these elements are simultaneously present. It will become clear that the transport of hydrogen and the transport of carbon are largely independent from each other, and thus this segmental approach is justified.

**Fig. 11:**
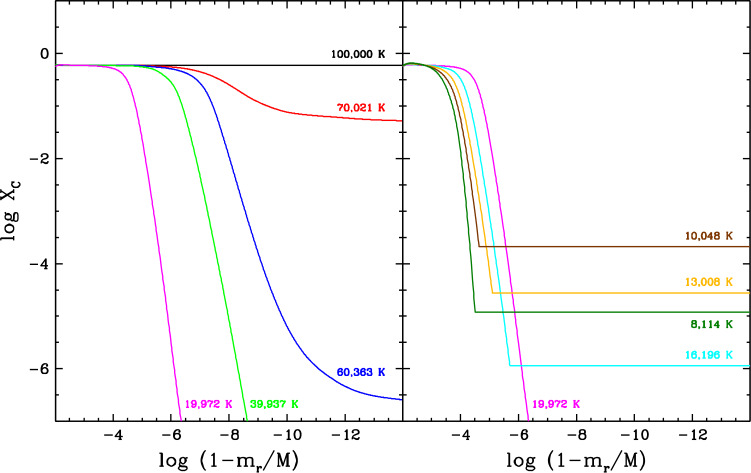
Carbon mass fraction profile at various effective temperatures in a typical simulation of element transport in helium-rich white dwarfs. The position in the star is measured in terms of the fraction of the total mass located outside a given radius, such that the surface is towards the right and the core is towards the left. This particular simulation is taken from Blouin et al. (2023a), and assumes a stellar mass of 0.55 $M_{\odot }$ and an initial carbon mass fraction $X_{\mathrm{C}} = 0.60$. The effective temperature decreases monotonically with time; the left panel illustrates the settling process at high temperature, while the right panel illustrates the convective dredge-up process at low temperature. In the latter case, notice the non-monotonic behaviour of the surface carbon abundance, which first increases (cyan, yellow, and brown curves) and then decreases (dark green curve)

The chemical evolution of a helium-, carbon-, and oxygen-rich progenitor is governed by the same three main physical mechanisms invoked in the last section: radiative wind, gravitational settling, and convection. To illustrate this, Fig. 11 shows the carbon abundance profile as a function of effective temperature in a simulation starting from a PG 1159-type composition (Bédard et al. 2022b; Blouin et al. 2023a). In the absence of processes opposing diffusion, the carbon and oxygen would be expected to sink out of sight within a few years. In reality, they are supported in the outer layers for much longer by the radiative wind. Indeed, the fact that PG 1159 stars are observed down to $T_{\mathrm{eff}} \simeq 75{,}000$ K is considered as another conclusive empirical proof of the existence of winds at the beginning of the cooling sequence (Unglaub and Bues 2000; Werner and Herwig 2006; Quirion et al. 2012; Bédard et al. 2022b,a). The left panel of Fig. 11 shows that as the wind fades, the carbon and oxygen rapidly sink into the star, thereby producing a growing pure-helium layer at the surface and accordingly changing the spectral character to DO and then DB (Dehner and Kawaler 1995; Fontaine and Brassard 2002; Althaus and Córsico 2004; Althaus et al. 2005b, 2009b; Camisassa et al. 2017; Bédard et al. 2022b,a). With further cooling, the convection zone appears and sharply expands downward at $T_{\mathrm{eff}} \simeq 20{,}000$ K (see the bottom panel of Fig. 7). The convective flows eventually catch up with the settling carbon and oxygen, which are thus efficiently dredged up to the surface. In practice, the dredged-up material is significantly richer in carbon than in oxygen, because the latter is less abundant in the progenitor to begin with and also sinks faster due do its higher atomic weight. For this reason, only carbon may become visible again, giving rise to a DQ white dwarf (Koester et al. 1982; Fontaine et al. 1984; Pelletier et al. 1986; MacDonald et al. 1998; Althaus and Córsico 2004; Althaus et al. 2005b; Dufour et al. 2005; Scóccola et al. 2006; Brassard et al. 2007; Camisassa et al. 2017; Koester et al. 2020; Bédard et al. 2022b,a; Blouin et al. 2023a). The right panel of Fig. 11 shows that the atmospheric carbon abundance gradually increases as the convective region expands and reaches farther into the deep carbon reservoir.^9^ Once the base of the convection zone stabilises at $T_{\mathrm{eff}} \simeq 10{,}000$ K, carbon sinks out of the convection zone and thus the surface abundance starts decreasing again (Pelletier et al. 1986; Dufour et al. 2005; Brassard et al. 2007; Bédard et al. 2022b,a; Blouin et al. 2023a).

The quantitative predictions of this scenario can be compared to observations of carbon-bearing white dwarfs, starting with the classical DQ stars. For instance, we can now explain why carbon pollution is detected almost exclusively in helium-dominated objects: their hydrogen-dominated counterparts do not have carbon-enriched progenitors and have much shallower convection zones. More importantly, we will see below that the expected rate of carbon depletion at $T_{\mathrm{eff}} \lesssim 10{,}000$ K is perfectly consistent with the diagonal sequence formed by the classical DQ stars in the temperature–abundance diagram (Fig. 5). Therefore, the existence of these objects can be unambiguously attributed to the convective dredge-up of carbon (Pelletier et al. 1986; Dufour et al. 2005; Koester and Knist 2006; Brassard et al. 2007; Coutu et al. 2019; Koester and Kepler 2019; Koester et al. 2020; Bédard et al. 2022b,a; Blouin et al. 2023a). Nevertheless, as with the dilution of hydrogen, the predicted carbon abundance depends sensitively on the efficiency of convective overshoot, which is essentially a free parameter and thus limits the predictive power of the models (Koester et al. 2020; Bédard et al. 2022a).

In this context, a reasonable approach is to turn the problem around, that is, to rely on the observed narrow DQ sequence to calibrate the extent of overshoot. The main complication in this procedure is that the amount of dredged-up carbon is also influenced by other parameters, most notably the stellar mass and the composition of the progenitor. More specifically, carbon contamination is predicted to be more significant when the mass is lower and the initial carbon content is higher (Pelletier et al. 1986; Bédard et al. 2022a; Blouin et al. 2023a). The question, then, is what values should be assumed for these parameters while adjusting overshoot to fit the theoretical and empirical carbon abundances. Fortunately, the masses of DQ white dwarfs and the carbon abundances of their PG 1159 progenitors are well constrained by independent observations. First, although the typical white dwarf mass is $\simeq 0.60\,M_{\odot}$, most cool DQ stars have inferred masses closer to $\simeq 0.55\,M_{\odot}$, hence this slightly lower value is likely more appropriate (Coutu et al. 2019; Bédard et al. 2022a; Caron et al. 2023). Second, as mentioned above, the PG 1159 population is characterised by $0.20 \lesssim X_{\mathrm{C}} \lesssim 0.60$, and it can be argued that the progenitors of DQ white dwarfs probably lie at the upper end of this range. Indeed, we have already remarked earlier that the observed DQ sequence sits just above the optical detection limit of carbon (Fig. 5), suggesting that DQ stars actually represent the most carbon-rich members of a larger carbon-polluted population (Weidemann and Koester 1995; Dufour et al. 2005; Bédard et al. 2022a).

**Fig. 12:**
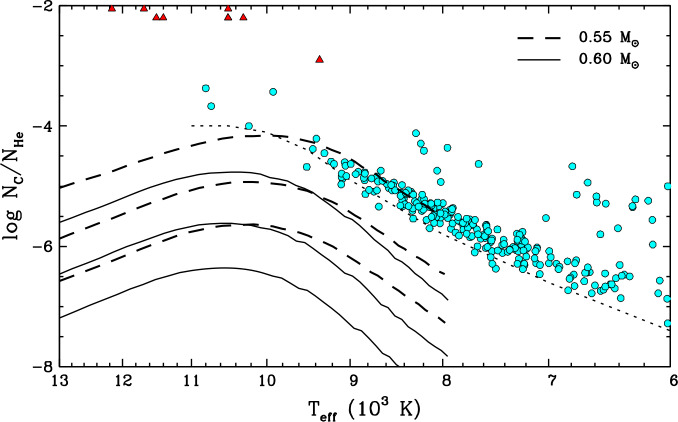
Zoomed-in version of Fig. 5 including theoretical predictions for the convective dredge-up of carbon. These simulations are taken from Blouin et al. (2023a), and assume stellar masses of 0.55 $M_{ \odot}$ (dashed curves) and 0.60 $M_{\odot}$ (solid curves) and initial carbon mass fractions $X_{\mathrm{C}} = 0.20$, 0.40, and 0.60 (from bottom to top)

Figure 12 presents a zoomed-in version of the temperature–abundance diagram including predictions from various transport simulations^10^ (Blouin et al. 2023a). The reference model assuming a mass of 0.55 $M_{\odot}$ along with the initial condition $X_{\mathrm{C}} = 0.60$ corresponds to the uppermost dashed line (this is the simulation shown in detail in Fig. 11). Of course, the perfect match to the empirical DQ sequence is the outcome of the fine-tuning of convective overshoot. Still, overshoot mainly impacts the height of the theoretical curve in the diagram, but affects its overall shape to a much lesser extent (Bédard et al. 2022a). Therefore, the fact that the slope of the predicted decrease at $T_{\mathrm{eff}} \lesssim 10{,}000$ K aligns with the DQ sequence constitutes an independent validation of the simulation, at least in this regime. Given this anchor point, the stellar mass and initial carbon content can then be varied to quantitatively assess their influence on the dredge-up process. The two lower dashed lines correspond to models with $X_{\mathrm{C}} = 0.40$ and 0.20 but still assuming 0.55 $M_{\odot}$. The set of solid curves uses the same three values for the initial carbon abundance while assuming a more standard mass of 0.60 $M_{\odot}$. As stated above, a higher mass and a less carbon-rich progenitor both translate into lighter carbon pollution at low effective temperature.

The results shown in Fig. 12 have three important implications. First, the fitting of the most carbon-polluted model on the observed DQ sequence provides a rough constraint on convective overshoot in cool ($T_{ \mathrm{eff}} \lesssim 10{,}000$ K) helium-rich white dwarfs. It is estimated that chemical mixing due to overshoot only extends over $\simeq 0.2$ pressure scale height, or $\simeq 0.1$ dex in $\log (1-m_{r}/M)$, below the convection zone.^11^ This distance is much smaller than that obtained from three-dimensional hydrodynamics calculations for hotter white dwarfs with shallower convection zones, indicating that overshoot becomes less significant as the convective region deepens. Second, the mass dependence of the dredge-up process naturally explains why classical DQ stars tend to have slightly lower-than-average masses. Carbon dredge-up likely occurs in most cool helium-dominated white dwarfs, but only those at the lower end of the mass distribution are polluted enough to show optical features earning them the DQ classification. Third, as DQ white dwarfs represent only the tip of the expected carbon abundance distribution at a given temperature, the vast majority of featureless DC white dwarfs should have unseen traces of carbon in their envelope (Bédard et al. 2022a).

The latter inference naturally takes us back to the topic of the Gaia bifurcation. We have seen in the previous section that the presence of hydrogen in most cool helium-rich white dwarfs is insufficient to explain the clear gap between the A and B branches. Nevertheless, the atmosphere of DC stars is predicted to contain not only hydrogen, but also carbon, which can further alter the spectral energy distribution. The right panel of Fig. 10 illustrates how the dredge-up of carbon impacts the course of helium-atmosphere white dwarfs in the Gaia colour–magnitude diagram; the three sequences displayed here assume evolving carbon abundances following the three 0.60 $M_{ \odot}$ simulations shown in Fig. 12. Unlike in the case of hydrogen, the successive increase and decrease of the atmospheric carbon content result in a significant deviation, such that the predicted curves satisfactorily coincide with the B branch. Accordingly, full population synthesis calculations taking carbon pollution into account successfully reproduce the separation between the A and B branches (Blouin et al. 2023a; Camisassa et al. 2023). Therefore, the Gaia bifurcation can be interpreted as the observational manifestation of the ubiquity of carbon dredge-up among the helium-rich white dwarf population. This remarkable conclusion has been independently corroborated by ultraviolet GALEX photometry, on which the opacity of carbon produces an even more distinctive signature (Blouin et al. 2023b). However, the agreement between the observed and predicted B branch is not perfect, which has been attributed to uncertainties in current models of convective dredge-up. In particular, the theoretical curves shown in Fig. 12 depend on the shape of the carbon diffusion tail at the bottom of the envelope, which is itself determined by the ionisation state of carbon at this depth (Pelletier et al. 1986; MacDonald et al. 1998; Blouin et al. 2023a). The equation of state of partially ionised carbon is thus an important model ingredient, yet it is currently poorly known, and this is especially problematic for the ascending part of the theoretical curves (Blouin et al. 2023a). Ultraviolet abundance measurements for cool DB white dwarfs with $15{,}000\,\mathrm{K} \gtrsim T_{\mathrm{eff}} \gtrsim 11{,}000$ K could provide useful empirical constraints on the rate of carbon enrichment.

Recently, Farihi et al. (2022) presented an alternative view of the origin of classical DQ white dwarfs, where these objects are products of binary stellar evolution. Among other arguments, they allege that the relatively low helium contents (or high carbon contents) and low stellar masses of DQ stars constitute evidence of a binary origin. This interpretation is at odds with the considerations of the present section. While it is true that DQ white dwarfs descend from carbon-enriched (hence moderately helium-deficient) progenitors, this is by no means a conundrum: such a PG 1159-type composition is the natural outcome of a well-known variant of single-star evolution, namely, the born-again scenario. This connection was first established in a seminal paper by Althaus et al. (2005b), who modelled the entire evolutionary channel from the zero-age main sequence, through the late helium-shell flash, and to the end of the cooling sequence. More recently, and as emphasised here, it has become clear that cool DQ stars are not fundamentally different from their DC and DZ counterparts, as both the DQ abundance distribution (Fig. 12) and the Gaia bifurcation (Fig. 10) indicate that most helium-atmosphere white dwarfs experience carbon dredge-up. Furthermore, we have already pointed out that the relatively low masses of DQ white dwarfs can be explained by the mass dependence of the convective dredge-up process^12^ (Fig. 12). It is beyond the scope of this review to examine the other arguments put forward by Farihi et al. (2022), but we note that some of them have been challenged by other works (Bagnulo and Landstreet 2022; Blouin 2022; O’Brien et al. 2023). All in all, the case for a binary origin appears inconclusive.

The convective dredge-up scenario nicely describes the atmospheric composition of the classical, cool DQ stars, but can it also explain the existence of other groups of carbon-bearing white dwarfs? Currently, this is not the case of the relatively hot, carbon-polluted DB stars with $T_{\mathrm{eff}} \simeq 25{,}000$ K and $\log N_{\mathrm{C}}/N_{\mathrm{He}} \simeq -5.5$ (Fig. 5). At this temperature, the convection zone is still very shallow (see the bottom panel of Fig. 7), hence standard transport simulations do not predict any surface carbon contamination. This suggests that the gravitational settling of carbon must be significantly slower than expected in these objects. However, none of the physical mechanisms usually invoked in the framework of spectral evolution provides a viable explanation for this behaviour. Therefore, the origin of carbon pollution in hot DB white dwarfs remains an open question (Fontaine and Brassard 2005; Koester et al. 2014b).

As for the massive DQ stars forming a second sequence in the temperature–abundance diagram (Fig. 5), it is clear that the convective dredge-up scenario does not apply in its canonical version, given that these objects originate from white dwarf mergers rather than single-star evolution (Dunlap and Clemens 2015; Cheng et al. 2019; Coutu et al. 2019; Kawka et al. 2023). Nevertheless, it has been demonstrated that the dredge-up process can indeed produce the required carbon-dominated atmospheric composition, provided that the stellar envelope is strongly helium-deficient (in addition to being strongly hydrogen-deficient, of course). More specifically, transport models indicate that the total helium mass must be between $\simeq 10^{-8}\,M$ and $10^{-6}\,M$ (Althaus et al. 2009a; Hollands et al. 2020; Koester et al. 2020), many orders of magnitude smaller than the value expected from single-star evolution, $\simeq 10^{-2}\,M$ (Iben and Tutukov 1984; Herwig et al. 1999; Lawlor and MacDonald 2006; Miller Bertolami and Althaus 2006; Renedo et al. 2010; Romero et al. 2012). Given such an initial condition, the surface helium layer built by diffusion remains extremely thin, and thus the expansion of the convection zone at $T_{\mathrm{eff}} \simeq 20{,}000$ K (see the bottom panel of Fig. 7) suddenly brings a large amount of carbon to the surface (Althaus et al. 2009a). The inferred strong helium deficiency represents yet another peculiar property of warm DQ stars (besides their high masses, space velocities, magnetic fields, and rotation rates) and provides valuable insight into the outcome of white dwarf mergers.

That said, we note that no single transport simulation has so far been able to reproduce the shape of the massive DQ sequence in the temperature–abundance diagram (Brassard et al. 2007; Althaus et al. 2009a). This may be because these objects have a highly non-standard cooling history, an aspect that is not considered in existing models as it has been uncovered only recently. In the Gaia colour–magnitude diagram, most massive DQ stars lie on the so-called Q branch, a nearly horizontal overdensity of white dwarfs at absolute magnitude $M_{G} \simeq 13$ (see Fig. 6; Gaia Collaboration et al. 2018a). This pile-up reveals that some white dwarf merger remnants experience a very long cooling delay of about 10 Gyr, which is likely due to a chemical distillation process triggered by the crystallisation of their core (Cheng et al. 2019; Blouin et al. 2021; Bédard et al. 2024). The warm DQ stars probably all belong to this delayed population, and therefore a given object is presumably stuck at a given effective temperature for billions of years. This means that the massive DQ sequence in the temperature–abundance diagram may not be an evolutionary sequence, and may instead arise from the fact that different objects experience the cooling delay at different temperatures (due to their different masses; Bédard et al. 2024). The spread in atmospheric composition could then simply reflect a diversity of residual hydrogen and helium contents and/or convection zone depths (Koester et al. 2020; Kilic et al. 2024). In any case, models of element transport over the multi-Gyr cooling delay are needed to shed further light on the chemical structure of merger remnants.

In concluding this section, we briefly consider the general (and more realistic) case of a helium-rich progenitor containing both a trace of hydrogen and a significant amount of carbon. The chemical evolution of such an object is simply a combination of the phenomena described in the previous and present sections. The surface composition initially remains unchanged because of the radiative wind, but as gravitational settling starts to operate, the star turns into a helium-atmosphere DO white dwarf due to the sinking of carbon, and then into a hydrogen-atmosphere DA white dwarf due to the floating of hydrogen. Shortly afterwards, the surface hydrogen layer is wiped out by convection, through either the convective dilution or mixing mechanism, thereby producing a DBA or helium-rich DA star. As the white dwarf cools further, the atmospheric hydrogen abundance remains roughly constant but its spectroscopic signature gradually disappears. Meanwhile, the convection zone reaches into the deep reservoir of settling carbon, a small amount of which is thus brought back to the surface. Depending on the magnitude of this dredge-up process, the outcome is either a DQ star if carbon is abundant enough to be spectroscopically detected, or a featureless DC star otherwise. In any case, it is clear that the resulting cool white dwarf does not possess a pure-helium atmosphere, but rather a helium-rich atmosphere contaminated by traces of both hydrogen and carbon (Bédard et al. 2022a).

## Conclusion

**Fig. 13:**
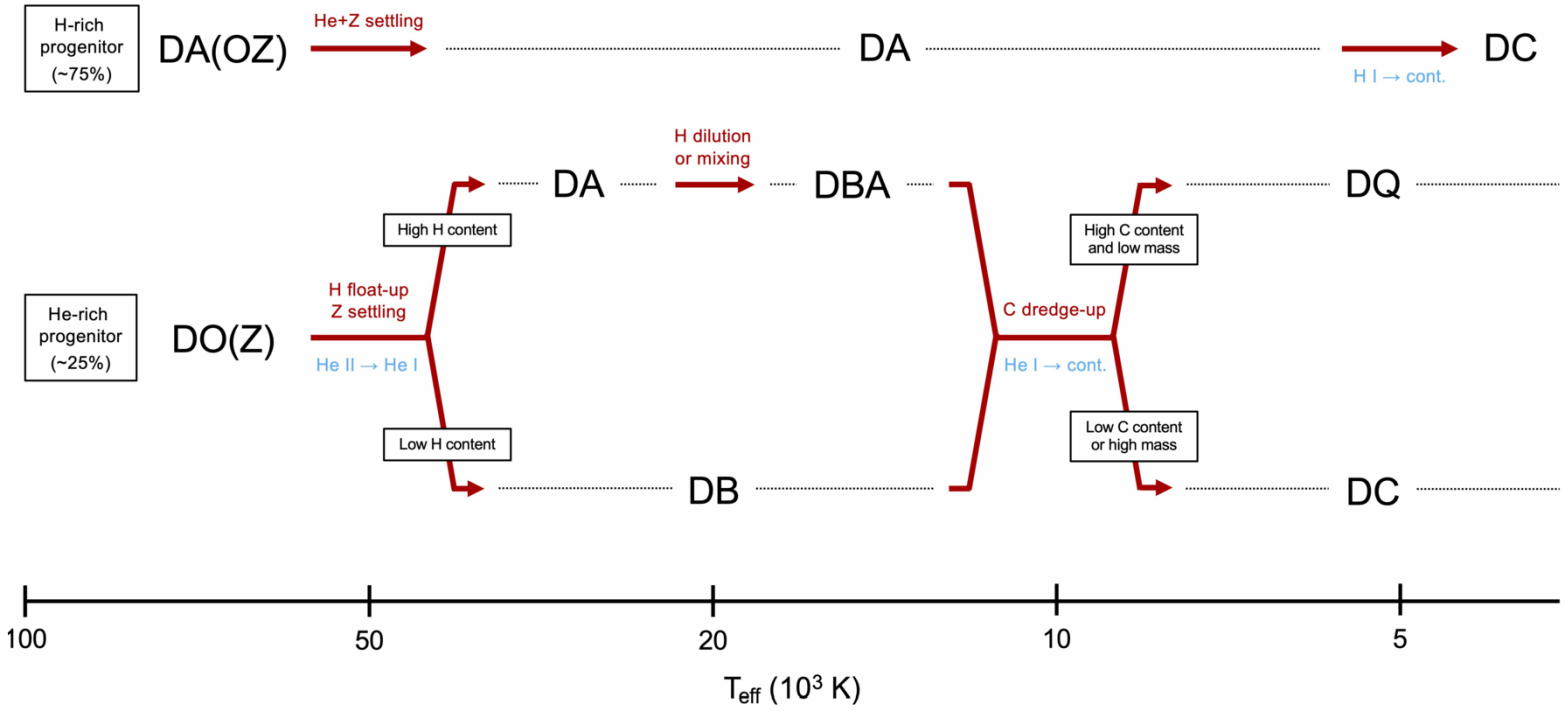
Schematic summary of the main white dwarf spectral evolution channels. For clarity, only internal element transport is considered, and it is implied that external accretion may at any time add metals to the atmosphere and thus add a “Z” to the spectral type. Some intermediate phases are also omitted for simplicity, most notably the hot stratified white dwarfs (amidst the DO-to-DA transition) and the cool helium-rich DA white dwarfs (amidst the DBA-to-DC/DQ transition)

Overall, our understanding of the spectral evolution of white dwarfs is in a very satisfactory state. On one hand, large-scale photometric and spectroscopic surveys have led to a detailed empirical view of the variations in surface composition along the cooling sequence. On the other hand, sophisticated numerical simulations of element transport have enabled the development of an elaborate theory of spectral evolution, which successfully explains nearly all of the observed features. As a summary, we present in Fig. 13 a schematic diagram of the main white dwarf spectral evolution channels as currently understood.

However, we still face a number of challenges, the most notable of which are briefly recapitulated here.

- The inferred fraction of helium-atmosphere white dwarfs remains uncertain, both at high temperature, where only one modern study exists, and at low temperature, where different studies yield different results. Better constraints would help clarify the relative importance of the three main spectral evolution channels among the white dwarf population.
- The undetected presence of hydrogen and carbon in cool helium-rich DC stars is supported by both observation and theory, but the abundances of individual objects remain elusive, which introduces a certain level of uncertainty in the determination of their atmospheric parameters. Ultraviolet spectroscopy, although currently practicable only for the nearest objects, could allow abundance measurements and hence greatly improve the characterisation of cool helium-dominated white dwarfs.
- More theoretical work is required to fully understand the transport of carbon in the envelope of white dwarfs. First, despite the overall success of the canonical dredge-up scenario, the quantitative predictions are still subject to uncertainties on the carbon equation of state, which thus needs to be improved. Second, this scenario currently completely fails to account for the carbon pollution seen in DB stars. Third, a holistic explanation for the peculiar atmospheric properties of the massive DQ class has yet to be provided.
- The spectral evolution of the hottest white dwarfs is believed to be dominated by radiative winds, and yet models describing the generation of such winds are practically nonexistent, forcing the use of very crude approximations in transport simulations. Detailed predictions of the mass-loss rate as a function of the relevant white dwarf parameters would be highly desirable.
- The predictive power of models of convectively-driven phenomena (namely, convective dilution, convective mixing, and convective dredge-up) is currently severely limited by uncertainties on the extent of overshoot. An expansion of the existing hydrodynamics-based overshoot studies could help reduce these uncertainties. Moreover, we note that the onset of the convective dilution process, where the helium-rich convection zone erodes the overlying hydrogen-rich layer from below, has yet to be modelled in detail. This may become possible with a more accurate description of overshoot.

In closing, we note that the advent of the next generation of multi-object spectroscopic surveys, including SDSS-V (Kollmeier et al. 2017), DESI (DESI Collaboration et al. 2016), WEAVE (Dalton et al. 2012), and 4MOST (de Jong et al. 2014), is imminent. This observational revolution will not only significantly increase the number of white dwarfs of all known types, but also possibly reveal entirely new classes of objects with atmospheric properties never seen before. These discoveries will constitute as many new challenges for the theory of white dwarf spectral evolution.

## Data Availability

No datasets were generated or analysed during the current study.

## Footnotes

1 In their original classification system, Sion et al. (1983) included an exception by which the DQ spectral type could be assigned based on the presence of carbon features not only in the optical, but in any part of the electromagnetic spectrum. We discourage the use of this exception, as it would create a two-tier classification system where the distinction between the DC and DQ spectral types would largely depend on the availability of an ultraviolet spectrum. As discussed later in this review, most optically featureless white dwarfs are expected to exhibit carbon features in the ultraviolet, however ultraviolet spectra are currently (and will likely remain) unavailable in the overwhelming majority of cases. We believe that these objects should be referred to as DC white dwarfs regardless of their ultraviolet features, so that the main spectral designation remains independent of sparsely available ultraviolet spectroscopy.

2 There are a few exceptions. First, a small fraction of DQ-type white dwarfs have a carbon-dominated atmosphere (see Sect. 2.4). Second, at $T_{\mathrm{eff}} \lesssim 5000$ K, the DZ class includes both hydrogen-rich and helium-rich atmospheres (since hydrogen lines in turn disappear at these temperatures).

3 In addition, other letters can be used to indicate the presence of emission features (“e”), a magnetic field (“H”), or photometric variability (“V”).

4 At $T_{\mathrm{eff}} \gtrsim 100{,}000$ K, the helium-rich fraction is actually much higher (80–90%; Bédard et al. 2020), but this is likely not representative of the true incidence of hydrogen-rich and helium-rich atmospheres. This is because helium-dominated white dwarfs cool much more slowly than their hydrogen-dominated counterparts in this early phase (Werner et al. 2019), and thus the effective temperature is not a good proxy for the cooling age.

5 Strictly speaking, “warm DQ” and “hot DQ” usually denote objects cooler and hotter than $T_{\mathrm{eff}} \simeq 18{,}000$ K, respectively, but the distinction is mostly historical.

6 On the other hand, metals other than carbon cannot be invoked to explain the location of the B branch, because the surface abundances required to alter the Gaia magnitudes would produce clearly visible calcium features (Blouin et al. 2023a).

7 A minority of helium-rich pre-white dwarfs show unexpectedly low carbon and oxygen abundances, corresponding to the O(He) spectral class; the origin of these objects is still debated (Rauch et al. 1998; Reindl et al. 2014b).

8 For numerical convenience, the transport simulation shown in Fig. 8 does not include the outer 10$^{-14} \,M$ of the star. As a consequence, the thickness of the surface hydrogen layer at $T_{\mathrm{eff}} \simeq 31{,}400$ K is overestimated and the onset of the convective dilution process cannot be modelled per se. This limitation is circumvented by inducing convective dilution artificially, which still provides a reliable prediction of the ensuing chemical evolution (see Bédard et al. 2023 for details).

9 Comparing the right panels of Figs. 8 and 11, it can be seen that the extent of the convectively mixed region, corresponding to the flat part of the abundance profiles, is not exactly the same at a given effective temperature. This is due to the assumption of different stellar masses and the use of different prescriptions for the efficiency of convective overshoot (Bédard et al. 2022b, 2023).

10 The model predictions shown in Fig. 12 do not extend below $T_{\mathrm{eff}} \simeq 8000$ K due to the lack of low-temperature carbon opacity data (Bédard et al. 2022a).

11 The constraint quoted in Bédard et al. (2022a), $\simeq 0.8$ pressure scale height, is different from that given here, $\simeq 0.2$ pressure scale height, as the latter is based on the improved models presented in Blouin et al. (2023a).

12 Farihi et al. (2022) further argue that most DQ stars have such low masses that their inferred total ages are larger than the age of the Galactic thin disk. However, this inference is highly uncertain as the main-sequence lifetimes of low-mass white dwarfs are notoriously poorly constrained (Heintz et al. 2022). For instance, had they used the initial-to-final mass relation of El-Badry et al. (2018) rather than that of Cummings et al. (2018), they would have found the vast majority of DQ white dwarfs to be consistent with single-star evolution. Besides, it is also possible that the absolute mass scale of DQ stars is currently slightly underestimated due to inaccuracies in atmosphere models (Coutu et al. 2019).
